# Computational NMR investigation of mixed-metal (Al,Sc)-MIL-53 and its phase transitions†

**DOI:** 10.1039/d3cp04147f

**Published:** 2023-09-22

**Authors:** Zachary H. Davis, Emma A. L. Borthwick, Russell E. Morris, Sharon E. Ashbrook

**Affiliations:** a School of Chemistry, EaStCHEM and Centre of Magnetic Resonance, University of St Andrews St Andrews KY16 9ST UK sema@st-andrews.ac.uk rem1@st-andrews.ac.uk

## Abstract

Compositionally complex metal–organic frameworks (MOFs) have properties that depend on local structure that is often difficult to characterise. In this paper a density functional theory (DFT) computational study of mixed-metal (Al,Sc)-MIL-53, a flexible MOF with several different forms, was used to calculate the relative energetics of these forms and to predict NMR parameters that can be used to evaluate whether solid-state NMR spectroscopy can be used to differentiate, identify and characterise the forms adopted by mixed-metal MOFs of different composition. The NMR parameters can also be correlated with structural features in the different forms, giving fundamental insight into the nature and origin of the interactions that affect nuclear spins. Given the complexity of advanced NMR experiments required, and the potential need for expensive and difficult isotopic enrichment, the computational work is invaluable in predicting which experiments and approaches are likely to give the most information on the disorder, local structure and pore forms of these mixed-metal MOFs.

## Abstract 2

DFT calculations are employed to gain insight into the structure of mixed-metal (Al,Sc)-MIL-53 and the potential of NMR spectroscopy to provide information on the composition, cation distribution and breathing behaviour of the framework.

## Graphical abstract

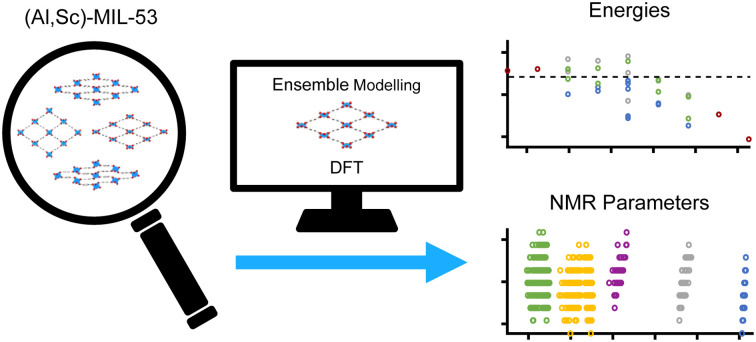

## Introduction

Metal–organic frameworks (MOFs) are a class of microporous materials composed of metal nodes and organic linkers.^1^ MOFs are recognised for their high surface areas and tuneable pore sizes, leading to potential applications in the fields of gas adsorption,^2–4^ separation,^5–7^ catalysis^8–11^ and healthcare.^12,13^ There is an increasing desire to better understand the structure–property relationship in MOFs, particularly for multivariate frameworks, such as mixed-metal MOFs, where there is the opportunity to tune their properties to target a specific application.^14,15^

**Fig. 1:**
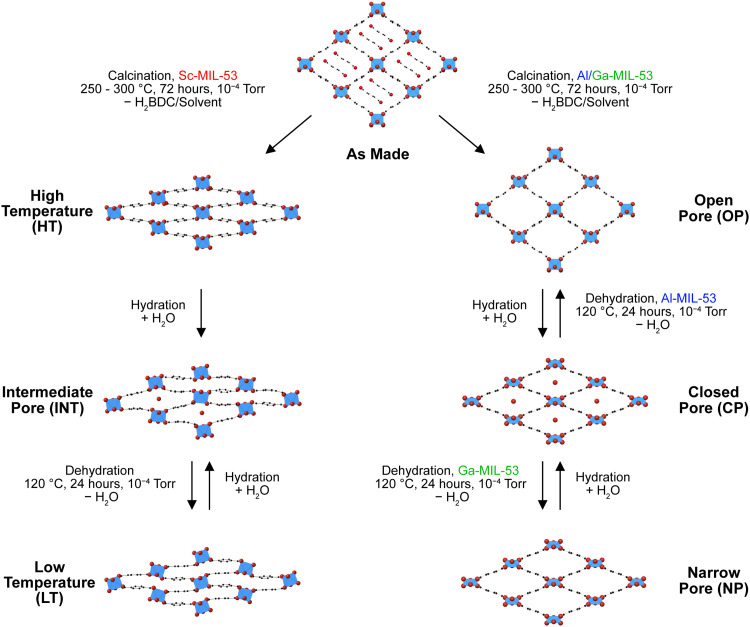
Schematic showing the breathing behaviour of Al-, Ga- and Sc-MIL-53 and the pore forms adopted by each.

MIL-53 (MIL = Matériaux de l’Institut Lavoisier) is a benzenedicarboxylate (BDC) based MOF, and was first synthesised under hydrothermal conditions by Serre *et al.* with Cr^3+^ as the metal node, giving Cr[OH]·[O_2_C–C_6_H_4_–CO_2_]·(H_2_O)_*y*_·(O_2_C–C_6_H_4_–CO_2_)_*y*_.^16^ Since this, MIL-53 has also been prepared with Al^3+^, Fe^3+^, In^3+^, Ga^3+^ and Sc^3+^.^17–21^ The structure of MIL-53 is composed of corner-sharing metal octahedra connected by μ_2_-OH groups. These chains of metal nodes are connected into layers by the organic linker, BDC^2−^, forming a 3D porous “wine-rack” like structure, as shown in Fig. 1. MIL-53 has been shown to exhibit large changes in its pore size depending upon the external conditions, such as temperature and/or pressure, and the type of guest molecules present. This so-called “breathing behaviour” is also influenced by the type of metal cation present in the framework.^22^ For example, as shown in Fig. 1, Al-MIL-53 adopts an open-pore (OP) form upon calcination (also referred to as activation).^17^ Subsequent cooling and exposure of the framework to the atmosphere results in the adsorption of H_2_O and the formation of a closed-pore (CP) form of the MOF. Removal of these guest H_2_O molecules results in the re-formation of the OP form, and (for Al-MIL-53) this hydration/dehydration process is fully reversible.^17^ The breathing behaviour of Sc-MIL-53 differs significantly forming a very-narrow-pore (VNP) structure, also sometimes known as the high-temperature (HT) form, when calcined (Fig. 1).^22,23^ Upon hydration, H_2_O is only found in half of the channels, with tilting of the ScO_6_ octahedra blocking access to the remaining pores. Dehydration affords a second type of CP form (which is different to the CP form observed for Al-MIL-53), sometimes referred to as the low-temperature (LT) form.^23^ Within this work the HT and LT naming convention is used when referring to these two Sc-MIL-53 pore forms, to differentiate these from the OP and CP forms of Al-MIL-53.

NMR spectroscopy has been shown to be a powerful tool for probing the structure of MOFs.^24–26^ The sensitivity to the local, atomic-scale environment of this approach means it is well suited to the study of materials exhibiting different levels or types of disorder.^27,28^ However, it can be challenging to interpret the complex and overlapped spectral lineshapes that result particularly for quadrupolar (*I* > 1/2) nuclei. In recent years interpretation has increasingly been achieved by combining experiment with computation in an approach often referred to as NMR crystallography.^29–31^ Our previous work used this approach to understand the effect of the cation composition on the overall breathing behaviour of mixed-metal (Al,Ga)-MIL-53.^32–34^^17^O NMR spectroscopy of isotopically enriched materials^32–34^ provided information on the framework composition through the relative intensities of the μ_2_-OH groups that bridge adjacent metal centres, and also showed a slight preference, relative to a random cation distribution, for clustering/pairing of like cations in the metal chains. We showed using ^13^C and ^17^O NMR spectroscopy that the breathing behaviour observed for the mixed-metal materials differs with composition, but that this could not be described as the compositionally weighted average of that for the two end members (see Fig. 1 for breathing behaviour for Ga-MIL-53), with mixed-pore materials seen, where OP and NP forms exist simultaneously.^33^ More recently, we exploited isotopic enrichment in different synthetic steps to show that post-synthetic modification of Al- or Ga-MIL-53 using ion-exchange resulted in materials that had a very similar overall composition to those made previously, but a very different structure, with a mixed-metal shell encasing a single-metal core.^34^

Here we aim to build on our previous work by considering the breathing behaviour of mixed-metal (Al,Sc)-MIL-53, which is likely to be more complicated given the additional and different pore forms exhibited by the Sc-containing end member (Fig. 1). The synthesis of (Al,Sc)-MIL-53 materials is likely to be more challenging given the different conditions typically used to produce Al- and Sc-MIL-53 (hydrothermal reaction in the first case and solvothermal reaction in the second),^17,23^ and the previous observation of other Sc-based MOFs, such as Sc_2_BDC_3_,^35^ either as major products or minor impurities.^32^ When this is considered alongside the very high cost of ^17^O isotopic enrichment (with 1 mL of 90% H_2_^17^O(l) costing ∼£3k),^26^ it is useful to first employ a computational approach to (i) explore the relative energies of the different possible pore forms and how these vary with composition, and (ii) predict the NMR parameters for each pore form. This will help to assign experimental NMR spectra in the future, and, perhaps more importantly, to evaluate the likely success (*i.e.*, the possible differentiation and resolution of different signals) of using solid-state NMR spectroscopy to determine framework composition and follow breathing behaviour in (Al,Sc)-MIL-53. From these data we will also be able to gain insight into the variation of the NMR parameters with local structure and the structural origin of the differences seen.

## Computational methods

First-principles DFT calculations were conducted using CASTEP versions 18.1 and 19.11 (see ESI†),^36–38^ carried out on either the Kennedy High-Performance Computing Cluster at the EaStCHEM computing facility at the University of St Andrews or the Young UK National Tier 2 High-Performance Computing Hub at University College, London. Kennedy consists of 110 computing nodes, each containing 32 cores (two 2.1 GHz 16-core Intel Broadwell (Xeon E5-2683 or Gold 6130) processors). All components are linked *via* FDR Infiniband and Gigabit Ethernet. Young is comprised of 576 computing nodes, each containing 40 cores (two 2.5 GHz 20-core Intel Xeon Gold 6248 processors). All calculations were performed using a planewave basis set, the GGA PBE^39^ exchange–correlation functional, ultrasoft pseudopotentials,^40^ ZORA relativistic treatment^41^ and the semi-empirical dispersion correction of Tkatchenko and Scheffler.^42^ Calculations were converged as far as possible with respect to both *k*-point spacing and energy cutoff and were carried out using a Monkhorst–Pack grid^43^ with *k*-point spacing of 0.04 2π Å^−1^ and a cutoff energy of 60 Ry (∼816 eV).

Structural models were generated for (Al_1−*x*_Sc_*x*_)-MIL-53 in the four pore forms (OP, NP, HT and LT) shown in Fig. 1 as described in Section S1 of the ESI.† The site-occupation disorder (SOD) program^44^ was used to generate the complete set of symmetrically inequivalent configurations for compositions with *x* = 0, 0.125, 0.25, 0.375, 0.5, 0.625, 0.75, 0.875 and 1. Configurations are equivalent if there is an isometric transformation that interconverts them; these transformations are the symmetry operations of space groups *Imma*, *P*2_1_/*c*, *C*2/*c* and *P*2_1_/*c* for OP, NP, HT and LT forms, respectively. The nomenclature used for each structure, their associated cation arrangement and the configurational entropy (or degeneracy) is provided in Table S1.1 of the ESI.† Each structure was subject to a geometry optimisation before the calculation of NMR parameters. Atomic coordinates and unit cell parameters were allowed to vary as part of the geometry optimisation, with an energy tolerance of 1 × 10^−4^ eV per atom and an electronic structure energy tolerance of 1 × 10^−9^ eV per atom used.

NMR calculations provide the absolute shielding tensor (**σ**) in the crystal frame. Diagonalisation of this gives the three principal components, *σ*_11_, *σ*_22_ and *σ*_33_, and the isotopic shielding, *σ*_iso_ = (1/3)Tr{**σ**^PAS^}. To facilitate future comparison with experiment the isotopic chemical shift, *δ*_iso_, is calculated using a reference shielding, *σ*_ref_, as described in the ESI.† The quadrupolar coupling constant, *C*_*Q*_ = *eQV*_*ZZ*_/*h*, and the asymmetry parameter, *η*_*Q*_ = (*V*_*XX*_ − *V*_*YY*_)/*V*_*ZZ*_, are calculated from the three principal components of the electric field gradient (EFG) tensor, **V**, where *Q* is the nuclear quadrupole moment, which was 2.86, −25.58, 146.6 and −220 mb for ^2^H, ^17^O, ^27^Al and ^45^Sc, respectively.^45^

## Results and discussion

**Fig. 2:**
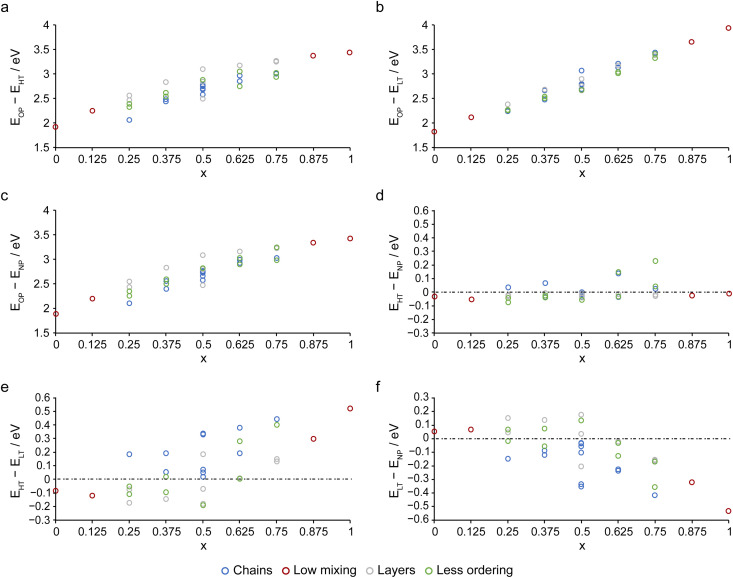
Plots showing the calculated energy differences between the four forms of (Al_1−*x*_Sc_*x*_)-MIL-53: (a) *E*_OP_ − *E*_HT_, (b) *E*_OP_ − *E*_LT_, (c) *E*_OP_ − *E*_NP_, (d) *E*_HT_ − *E*_NP_, (e) *E*_HT_ − *E*_LT_ and (f) *E*_LT_ − *E*_NP_ as a function of cation composition, coloured by the type of ordering.

Calculations were performed for every symmetry inequivalent combination of cations in the OP, NP, HT and LT forms of (Al_1−*x*_Sc_*x*_)-MIL-53 as described in Section S1 of the ESI,† on unit cells containing 8 cations (after transformation to ensure the definition of the unit cell axes and the numbering of the cation sites were comparable between the four forms). The (electronic) energy differences for a given cation arrangement between the different pore types are shown in Fig. 2. Note structures for the NP and LT forms have been grouped by their equivalent parent structure (*i.e.*, the equivalent OP and HT cation arrangement when removing symmetry restraints from the given NP or LT unit cell, as reported in Table S1.1 in the ESI†), and the average energy of these groups plotted. The cation arrangements have been assigned to one of four categories, as described in Table S1.1 in the ESI†: structures with levels of low mixing (E, red), chains (C, blue), layers (L, grey) and structures with overall less ordering (O, green). It should also be noted both that (i) the absolute energies calculated will depend on the functional used and (ii) the pore form(s) adopted experimentally will depend on the synthetic route and particularly the heat treatment applied. While absolute values should be treated with caution, trends across a series, and the relative magnitudes of differences are reasonable to interpret.

Fig. 2(a)–(c) show that for all cation combinations the OP form is energetically disfavoured over all other structures (*i.e.*, HT, LT and NP, respectively). However, the difference in energy between the OP form and other forms decreases with higher Al^3+^ content (*i.e.*, towards *x* = 0). A similar result was previously observed by Rice *et al.*^33^ for OP and NP (Al,Ga)-MIL-53, but it was shown that this was only the case when dispersion corrections were included in the DFT calculations. Without such corrections the OP form was favoured over the NP form for all compositions. Although dispersion corrections have been included in all calculations in this work, the differences seen by Rice *et al.* highlight the importance of including these accurately and the possible changes (both in the absolute values and in the resulting conclusions) that would be observed for under or overestimation of these in any chosen methodology. Comparing the HT and NP forms, as in Fig. 2(d), indicates overall a slight energetic preference for the NP structure, with some cation arrangements preferring the HT structure. There is no systematic change in this preference with cation composition over the series indicating that the HT and NP forms are energetically very similar. Both the HT and NP forms are favoured over the LT form when most of the cations present are Al^3+^ (as shown in Fig. 2(e) for the HT form). At *x* = 0.5, this changes and the LT form is predicted to be more stable than both the NP and HT structures with increasing amounts of Sc^3+^, as shown in Fig. 2(e) and (f). Noticeably, structures where the substituted cations lie in chains (blue), *i.e.*, with X–O(H)–X rather than X–O(H)–Y linkages lying along the *x* axis, favour adoption of the LT form compared to those with layers (grey) and less ordering (green). However, it should also be noted that the smaller size of the unit cell used to ensure that calculations and the number of unique configurations remain tractable result in high levels of ordering along the *x* axis, with only type of cation (*e.g.*, Al–O(H)–Al–O(H)–Al or Sc–O(H)–Sc–O(H)–Sc), shown in blue in Fig. 2, or alternating Al^3+^ and Sc^3+^ cations (*i.e.*, Al–O(H)–Sc–O(H)–Al).

For all four pore forms an increase in the unit cell volume is observed as the percentage of Sc^3+^ increases, as shown in Fig. S4.1 of the ESI,† as expected because of the size difference between the two cations (Shannon ionic radii of 0.745 Å for six-coordinate Sc^3+^ and 0.535 Å for six-coordinate Al^3+^).^46^ The increase is most significant for the OP structure, which expands by 526 Å^3^ between Al- and Sc-MIL-53. Equivalent changes for the NP, HT and LT forms are 80 Å^3^, 83 Å^3^ and 81 Å^3^, respectively. An increase in unit cell volume was also seen in previous work for OP (Al,Ga)-MIL-53,^33^ although the changes were smaller in magnitude (190 Å^3^), reflecting the smaller increase in the Shannon ionic radii for six-coordinate Ga^3+^ (0.62 Å) relative to Al^3+^. Notably, a much smaller change in unit cell volume was observed for the NP form of (Al,Sc)-MIL-53. There is some variation in the volume for a given composition depending on the cation arrangement, and this is noticeably larger than that observed previously for (Al,Ga)-MIL-53,^33^ reflecting again the smaller radius change between Al^3+^ and Ga^3+^ (*i.e.*, a difference of 0.085 Å rather than the 0.21 Å for Al^3+^ and Sc^3+^). It should be noted that one of the OP structures (Sc1358, where Sc^3+^ is located on cation sites 1, 3, 5 and 8, and all others contain Al^3+^), converged towards the NP form during the geometry optimisation step. This structure was re-optimised with a fixed unit cell size, after which a second optimisation was performed where the unit cell parameters were then allowed to change. The OP form was retained using this procedure. The small number of OP structures which have an overall smaller unit cell volume (*e.g.*, for *x* = 0.25 and 0.5) may well have started to contract in a similar manner but have not fully converted to the NP form, resulting in larger energy differences than perhaps expected. This observation indicates, once again, the stability of the smaller pore forms over that of the OP form when dispersion corrections are included in the geometry optimisation step. It is possible that re-optimising these structures using the above approach would result in an overall larger OP unit cell size.

The calculated mixing enthalpies, *E*_mix_, given by

**Fig. 3:**
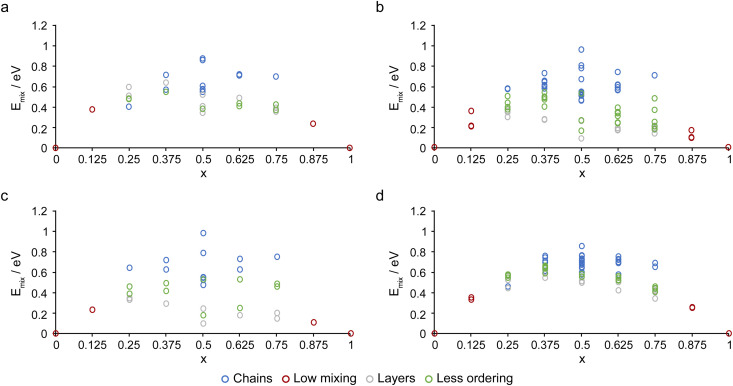
Plots of the relative mixing energies, *E*_mix_, for the (a) OP, (b) NP, (c) HT and (d) LT forms of (Al_1−*x*_Sc_*x*_)-MIL-53, as a function of cation composition, coloured by type of ordering.

1 *E*_mix_ = *E*(*x*_*i*_) − ((*x*_1_ − *x*_*i*_)*E*(*x*_0_) + *x*_*i*_*E*(*x*_1_)),

where *x*_0_ = 0 and *x*_1_ = 1, are shown in Fig. 3. Here, a negative *E*_mix_ value indicates that it is (in principle at least) energetically favourable to form the mixed-metal MOF, rather than a two-phase mixture of the single-metal end members. Fig. 3 shows all mixed-metal materials have a positive *E*_mix_ value suggesting it is enthalpically unfavourable to form these frameworks (and these values are notably larger than those seen previously for OP and NP (Al,Ga)-MIL-53, which were between −0.2 and +0.1 eV).^33^ However, these calculations do not consider entropy contributions, which should be favourable for most mixed-metal materials. Despite the positive values, *E*_mix_ is generally smaller for the NP and HT forms, with some values approaching zero for *x* = 0.5. In addition, ordering of like cations into layers (grey) appears energetically more favourable than chains (blue), with structural models showing less ordering (green) appearing in between. A similar result, although perhaps one that was not quite so clear, was reported for (Al,Ga)-MIL-53.^33^ It should also be noted that, as described above, the models used in this work allow only two arrangements of cations along the x axis, with chains of like cations (*i.e.*, all Sc^3+^ or all Al^3+^) or chains of alternating Al^3+^ and Sc^3+^ cations. It is possible that more disordered arrangements along these chains would be more favourable entropically.

The positive mixing enthalpies predicted here may explain some of the difficulties that have been seen in the experimental synthesis of (Al,Sc)-MIL-53, with direct synthesis in H_2_O resulting in a two-phase mixture of Al-MIL-53 and Sc_2_BDC_3_.^32^ In addition, however, the standard approaches for the synthesis of pure Al- and Sc-MIL-53 typically use different solvents, H_2_O and DMF, respectively, and synthesis of Sc-MIL-53 uses a base, often pyridine, to control the pH, potentially making it challenging to find ideal synthetic conditions for the formation of (Al,Sc)-MIL-53.^17,23^ It should also be noted that MOFs are not prepared in the calcined forms considered above but in their as-made forms, as shown in Fig. 1, *i.e.*, with excess linker and residual solvent within the pores. It will be the relative mixing energies in these forms (along with various kinetic considerations) that would ultimately determine whether mixed-metal materials could be easily, or even successfully, synthesised, and such a study is outside the scope of the current work. However, the more significant differences in energy seen in this work suggest that the preparation of mixed-metal (Al,Sc)-MIL-53 frameworks may well be synthetically more challenging. Plots of degeneracy-weighted *E*_mix_ values for a given composition of OP, NP, HT and LT (Al_1−*x*_Sc_*x*_)-MIL-53 are shown in Fig. S5.1 of the ESI.† These plots are not symmetric and show lower *E*_mix_ values at higher values of *x* for all four forms. This suggests substitution of a small amount of Al^3+^ into Sc-MIL-53 is generally energetically more favourable than substituting Sc^3+^ into Al-MIL-53 and indicates it may be preferential to attempt future experimental synthesis of (Al,Sc)-MIL-53 initially with a low Al:Sc ratio.

**Fig. 4:**
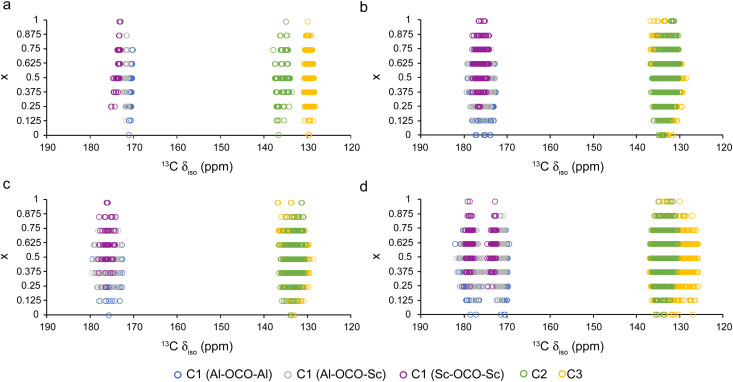
Plots showing calculated ^13^C *δ*_iso_ for (a) OP, (b) NP, (c) HT and (d) LT (Al_1−*x*_Sc_*x*_)-MIL-53 for different compositions, coloured by type of carbon.

As reported previously, ^13^C CP MAS NMR spectra can be used to differentiate between the various pore forms of (Al,Ga)-MIL-53 based on the chemical shift of the carboxylate carbon (C1), which appears at *δ* = 171 ppm for the OP form and 175 ppm for the NP form.^33^ The calculated ^13^C *δ*_iso_ values for the (Al_1−*x*_Sc_*x*_)-MIL-53 materials considered are shown in Fig. 4. For the OP form, three distinct C environments are seen: C1 (carboxylate carbon), C2 (ipso-carbon) and C3 (CH carbon). For C1, three sets of signals are seen for carboxylate groups bonded to different combinations of metal cations, with an increase in *δ*_iso_ with an increasing number of Sc^3+^ neighbours. However, these signals are unlikely to be resolved experimentally given the small differences in *δ*_iso_ and the typical line broadening in solid-state NMR spectra. The NP, HT and LT forms all show an increased range of *δ*_iso_ values for a particular type of C, with signals from the C2 and C3 environments overlapping significantly, especially for the NP and LT structures. This is not unexpected given the reduction in symmetry from the parent OP form to the NP, HT and LT structures, leading to an increased number of different of environments. The similar *δ*_iso_ values for a given type of ^13^C environment in all four of these forms might make it difficult to determine experimentally which pore form a framework has adopted using ^13^C NMR spectroscopy alone, particularly for the NP, LT and HT forms. It is possible that the OP form could be identified from the lower chemical shifts predicted for C1 and the clear separation of the C2 and C3 resonances. Interestingly, there are two unique groups of C1 chemical shifts for the LT form regardless of the adjacent cations. These differences arise due to the tilting of the MO_6_ octahedra within the structure, resulting in a variation of the O–C1–O angle, as shown in Fig. S6.1 of the ESI.† When *x* = 1 (*i.e.*, Sc-MIL-53), the C1 environment with the smallest O–C1–O angle (123.39°) has a higher calculated ^13^C *δ*_iso_ (178 ppm) compared with the C1 environment with the larger angle (124.28°) where the ^13^C *δ*_iso_ is 172 ppm. The presence of these two C1 signals may also allow structures adopting the LT form to be identified experimentally.

The calculated ^1^H *δ*_iso_ values, shown in Fig. S6.2 of the ESI,† indicate that the different μ_2_-OH groups would be resolved in a ^1^H MAS spectrum (assuming sufficiently rapid MAS was possible); however, the shift range for the Sc–O(H)–Sc site overlaps with that from the aromatic ^1^H on the BDC^2−^ linker in the NP, HT and LT forms. A larger distribution of *δ*_iso_ values is observed for NP, HT and LT forms because of the reduction in symmetry compared to the OP form, as observed previously for the calculated ^13^C *δ*_iso_ values. Experimentally, the ^1^H signals for the μ_2_-OH groups in calcined dehydrated Sc-MIL-53 are observed at lower shift than the ∼6 ppm suggested by the DFT calculations, indicating some inaccuracy in the absolute predicted ^1^H *δ*_iso_ values for the OH groups, or an additional effect from incomplete removal of water (and hydrogen bonding) or as a result of dynamics.^19^ Given this, it is perhaps unlikely that individual μ_2_-OH groups would be resolved in ^1^H NMR spectra of (Al,Sc)-MIL-53, especially at slower MAS rates, as is the case for (Al,Ga)-MIL-53,^33^ although it may be interesting to attempt very rapid MAS or variable temperature measurements on samples that are completely dehydrated to verify this. Another alternative would be to post-synthetically exchange the OH species for OD groups (with the corresponding ^2^H MAS NMR spectrum then avoiding overlap with the aromatic signals). Furthermore, ^2^H NMR spectra exhibit much smaller dipolar couplings, providing better resolution at slower MAS rates, and are typically very sensitive to different types and rates of motion/exchange if present. More generally, ^2^H spectra can provide further insight into hydrogen bonding through changes in *C*_*Q*_,^27,47^ which may also be of use when considering hydrated forms of MIL-53. A plot of calculated ^2^H *δ*_iso_*versus C*_*Q*_ for all four pore forms of the calcined materials is given in Fig. S6.3 of the ESI,† and shows different ^2^H *C*_*Q*_ values for the OD groups (∼0.28 MHz) and CD sites in the BDC^2−^ linker (∼0.18 MHz). The *C*_*Q*_ values predicted for three different types of OD groups are similar, perhaps as expected for dehydrated materials, although for each of the three there is a correlation with chemical shift for the NP, HT and LT forms.

**Fig. 5:**
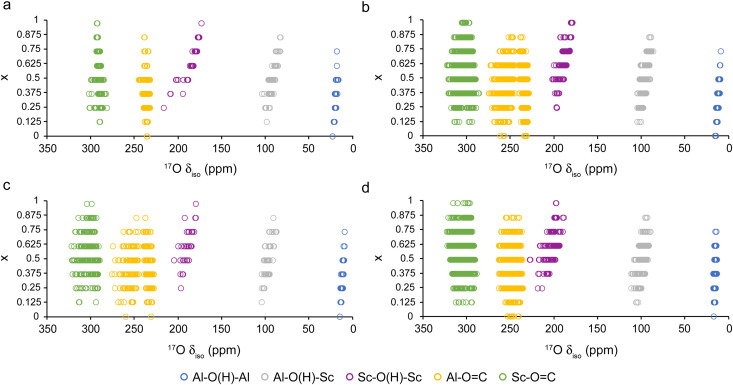
Plots showing calculated ^17^O *δ*_iso_ for (a) OP, (b) NP, (c) HT and (d) LT (Al_1−*x*_Sc_*x*_)-MIL-53 for different compositions, coloured by type of oxygen.

We have shown previously the utility of ^17^O NMR spectroscopy for providing structural information on (Al,Ga)-MIL-53 materials,^32–34^ with the number of carboxylate oxygen signals and their NMR parameters of the carboxylate oxygens differing between the OP and NP forms, and the relative intensity of the different types of μ_2_-OH sites providing information on the framework composition and the cation distribution. The calculated ^17^O *δ*_iso_ values for (Al_1−*x*_Sc_*x*_)-MIL-53 are shown in Fig. 5, plotted as a function of composition. Three distinct ranges of chemical shift are observed for the three μ_2_-OH groups, coloured in blue (Al–O(H)–Al), grey (Al–O(H)–Sc) and purple (Sc–O(H)–Sc). The predicted shifts for Al–O(H)–Al are very similar to those observed in (Al,Ga)-MIL-53 at ∼20 ppm.^32–34^ Shifts from OH groups bonded to Sc^3+^ are at higher *δ*_iso_ values, with Sc–O(H)–Al at ∼100 ppm and Sc–O(H)–Sc at ∼180 ppm. These values are much higher than the μ_2_-OH groups bonded to Ga^3+^ seen in previous work,^32–34^ which appear at ∼25 ppm and ∼30 ppm for Al–O(H)–Ga and Ga–O(H)–Ga environments, respectively. Work reported previously showed a ^17^O chemical shift of 143(4) ppm for Sc–O(H)–Sc groups in hydrated Sc-MIL-53 (*i.e.*, the intermediate, or INT, pore form shown in Fig. 1),^32^ enriched in ^17^O using a post-synthetic steaming method, which agrees reasonably well with the predictions of much higher calculated *δ*_iso_ values here (although some differences will be expected due to the different pore form and the presence of water). The large differences predicted in the calculated ^17^O *δ*_iso_ for the μ_2_-OH groups suggest these may not overlap significantly in ^17^O MAS NMR spectra at moderate to high field strengths (*e.g.*, 14.1–20.0 T), even when considering the quadrupolar broadening, unlike the case of (Al,Ga)-MIL-53.^32–34^ Thus, it is possible that information on the relative proportions of each OH group present could be obtained without the need for (non-quantitative and lengthy) MQMAS^48^ experiments to remove the quadrupolar interaction, enabling information on the cation distribution to be more easily obtained. A more significant correlation is observed between the ^17^O *δ*_iso_ for the Sc–O(H)–Sc species and the composition, with lower *δ*_iso_ seen with increasing Sc^3+^ content (*i.e.*, at larger values of *x*). This correlation arises from a variation in the Sc–O–Sc bond angle over the compositional series. Fig. S6.4 in the ESI† plots ^17^O *δ*_iso_ (for Sc–O(H)–Sc sites) against the Sc–O–Sc bond angle for the OP form of the MOF, in which a clear trend is observed. Smaller Sc–O–Sc angles are found in (Al_1−*x*_Sc_*x*_)-MIL-53 structures with lower values of x, resulting in higher ^17^O *δ*_iso_. It is likely that the large size difference between the Al^3+^ and Sc^3+^ cations results in steric strain, that increases the average Sc–O–Sc bond angle and, subsequently, decreases *δ*_iso_.

Two distinct sets of ^17^O *δ*_iso_ values are seen for each of the carboxylate signals (*i.e.*, for each of X–O=C, where X is Al or Sc) in the NP and HT forms of MIL-53. These result from similar structural changes to those seen previously for (Al,Ga)-MIL-53, where rotation of the BDC^2−^ linker results in different distances between the ^17^O carboxylate species and the μ_2_-OH group across the pore, creating two distinct carboxylate oxygen environments even in the end member.^32^ However, the position of these signals in a MQMAS spectrum (and the width of the lines in the MAS spectrum) also depends on the magnitude of the quadrupolar interaction, *C*_*Q*_. Fig. S6.5 in the ESI† shows a plot of calculated ^17^O *C*_*Q*_ against *δ*_iso_, where these two types of environments are now differentiated more clearly for the NP and HT forms by their NMR parameters. For carboxylate oxygens bound to Sc^3+^, there are small differences in both *δ*_iso_ and *C*_*Q*_ (with one set having a lower *δ*_iso_ and slightly higher *C*_*Q*_ than the second). A similar effect is seen for carboxylate oxygens bound to Al^3+^ in the NP and HT forms, although the magnitude of the differences is much greater between the two. Fig. S6.5d (ESI†) shows three sets of ^17^O carboxylate species (for each X–O=C) for the LT form, which originate from the tilting of the MO_6_ octahedra within the structure (as shown previously in Fig. S6.1, ESI†), altering the geometry around the ^17^O carboxylate sites. The ranges seen for the ^17^O *δ*_iso_ and *C*_*Q*_ values and the variation in several structural parameters with different atomic arrangements makes it difficult to ascertain unambiguously the exact origin of the differences. Fig. S6.6 in the ESI† shows that for Sc–O=C groups in LT (Al_1−*x*_Sc_*x*_)-MIL-53 there is perhaps a stronger dependence on the O–Sc bond length than the Sc–O=C bond angle, but neither effect dominates. No direct correlation with composition or type of cation arrangement appears to be present. However, when considering end member Sc-MIL-53 (*i.e.*, *x* = 1) eight unique ^17^O carboxylate species are present and can be differentiated based on their NMR parameters (*δ*_iso_ and *C*_*Q*_) which depend on the distance between the carboxylate oxygen and the hydrogen atom of the OH group across the pore, as shown in Fig. S6.7 in the ESI.† These species are grouped into three sets (containing two, four and two of the eight, respectively). The variation in distance to the OH group across the pore arises from the tilting of the MO_6_ octahedra described above. The additional disorder present when considering the incorporation of Al^3+^ into the framework results in a distribution of NMR parameters for each of these species, and thus only three groups of ^17^O carboxylate species are observed in Fig. S6.6d (ESI†). This is shown more clearly when once again considering the distance between the carboxylate oxygen and the hydrogen atom of the OH group across the pore as shown in Fig. S6.8 in the ESI.†

**Fig. 6:**
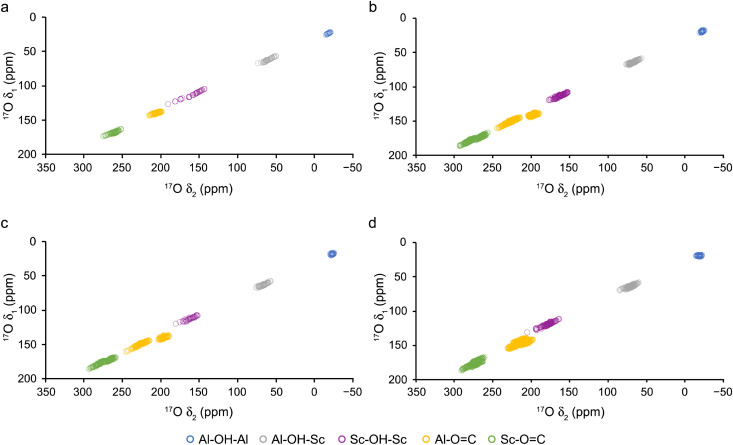
Plot of calculated ^17^O *δ*_1_ and *δ*_2_ positions of the centre-of-gravity of a resonance in a 20.0 T 3QMAS spectrum (after shearing) for (a) OP, (b) NP, (c) HT and (d) LT (Al_1−*x*_Sc_*x*_)-MIL-53.

The predicted centre-of-gravity of a resonance in a MQMAS spectrum for a spin *I* = 5/2 nucleus, *i.e.*, (*δ*_1_, *δ*_2_) can be calculated^27,48,49^ (at a specified magnetic field) from *C*_*Q*_ and *δ*_iso_, using eqn (S7.1)–(S7.4) in the ESI,† and the convention for referencing *δ*_1_ described in ref. 49. Fig. 6 plots predicted *δ*_1_ and *δ*_2_ positions (for 20.0 T ^17^O 3QMAS spectra, after shearing) for all four pore forms of (Al_1−*x*_Sc_*x*_)-MIL-53. Carboxylate oxygens bonded to Al^3+^ and Sc^3+^ are expected to be reasonably well resolved for all four pore forms (although some of these signals would overlap if different pore forms were present simultaneously in a sample). The presence of two types of chemically different carboxylate group (bonded to either Al^3+^ or Sc^3+^) can also be seen for the NP and HT forms, although this is more significant for carboxylate oxygens bonded to Al^3+^. Unfortunately, there is little resolution of the three different types of carboxylate oxygens in the LT form (*i.e.*, three for the carboxylates bound to Al^3+^ and three for those bound to Sc^3+^) at this field. As the differences in the NMR parameters for these species are primarily in *C*_*Q*_, better isotropic resolution is likely to be obtained at lower magnetic field, although this would be accompanied by poorer inherent sensitivity and broader spectral lines in *δ*_2_.^27^ However, good resolution of most O signals is predicted to occur at 20.0 T.

Fig. S6.9 and S6.10 in the ESI† show the predicted ^27^Al and ^45^Sc *δ*_iso_ values, respectively, for different compositions of (Al_1−*x*_Sc_*x*_)-MIL-53 for each of the four pore forms. Two chemically different cations environments are seen depending on the adjacent metal cations in the chain (*i.e.*, Al–O(H)–X–O(H)–Al and Sc–O(H)–X–O(H)–Sc). This distinction is clearest when the framework adopts the NP and HT forms. The shift differences are small for ^27^Al (2–4 ppm, Fig. S6.9, ESI†) but larger for ^45^Sc (∼20–30 ppm, Fig. S6.10, ESI†), although whether the two types of cations can be resolved experimentally would depend on the magnetic field used and the quadrupolar broadening (both its absolute magnitude and the relative difference in *C*_*Q*_ between the two – see later). However, it should also be noted that the imposed ordering from the single unit cell used discussed above produces two nearest neighbours that are the same, and it is likely Al–O(H)–X–O(H)–Sc environments will also be present, with chemical intuition suggesting the chemical shifts for these will lie somewhere in between the two sets of signals seen in each case, perhaps further limiting resolution at all but the highest fields.

**Fig. 7:**
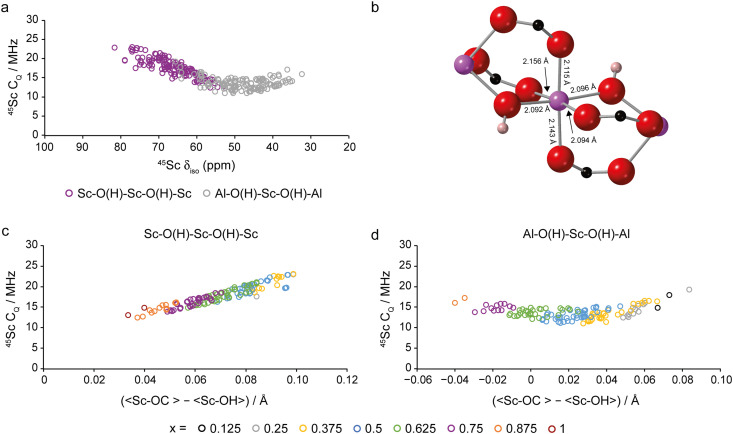
(a) Plot of calculated ^45^Sc *C*_*Q*_ against *δ*_iso_ for the LT for of (Al_1−*x*_Sc_*x*_)-MIL-53, coloured by type of next nearest neighbouring metals. (b) ScO_6_ octahedron in Sc-MIL-53, with Sc–O bond distances labelled. (c) and (d) Plots of calculated ^45^Sc *C*_*Q*_ as a function of the difference in the average Sc–OH and Sc–OC bond lengths for the ScO_6_ octahedra in LT (Al_1−*x*_Sc_*x*_)-MIL-53 for (c) Sc–O(H)–Sc–O(H)–Sc and (d) Al–O(H)–Sc–O(H)–Al. Points are coloured by composition of the overall framework.

Fig. S6.11 and S6.12 in the ESI† show plots of *C*_*Q*_ against *δ*_iso_ for ^27^Al and ^45^Sc, respectively, for all four pore forms of (Al_1−*x*_Sc_*x*_)-MIL-53. For ^45^Sc (Fig. S6.12, ESI†), there is an interesting correlation between *δ*_iso_ and *C*_*Q*_ in all cases, but this is most evident for the OP and LT forms. This is shown in Fig. 7(a) for the LT form. Between *δ*_iso_ values of 80 ppm and 50 ppm a decrease in *δ*_iso_ is accompanied by a decrease in *C*_*Q*_. However, below 50 ppm, *C*_*Q*_ begins to increase despite further decreases in *δ*_iso_. The Sc^3+^ is six-coordinate, with the ScO_6_ octahedron being composed of four bonds to carboxylate oxygens and two to (trans) μ_2_-OH groups, as shown in Fig. 7(b) for the Sc-MIL-53 end member. It can be shown that the ^45^Sc *C*_*Q*_ and *δ*_iso_ in the LT form depend on the difference between the average Sc–OH and Sc–OC bond lengths (*i.e.*, 〈Sc–OC〉–〈Sc–OH〉) in the ScO_6_ octahedron. This is shown for the ^45^Sc *C*_*Q*_ of the Sc–O(H)–Sc–O(H)–Sc and Al–O(H)–Sc–O(H)–Al species in Fig. 7(c) and (d), respectively. (See Fig. S6.13 in the ESI† for the corresponding plots for ^45^Sc *δ*_iso_.) For Sc-MIL-53 (*x* = 1), which can be viewed as the “ideal” structure, this difference is 0.037 Å (as seen in Fig. 7(b)), reflecting the generally shorter Sc–OH bonds. A deviation away from this value results in an increase in *C*_*Q*_ (*i.e.*, greater levels of “distortion”). Fig. S6.14 in the ESI† shows these changes are primarily a result of the variation in 〈Sc–OH〉, which shows a much larger range (by a factor of ∼2) over the compositional series than 〈Sc–OC〉, particularly for Al–O(H)–Sc–O(H)–Al. For Sc–O(H)–Sc–O(H)–Sc, an increase in the difference between the average bond lengths is observed with increasing amounts of Al^3+^ (Fig. 7(c)), which in turn results in larger ^45^Sc *C*_*Q*_ values. For Al–O(H)–Sc–O(H)–Al (Fig. 7(d)), difference values of close to the ideal value of 0.037 Å are found for compositions between *x* = 0.35 and 0.5. Both an increase and decease in *x* then results in greater distortion and an increase in ^45^Sc *C*_*Q*_ (due to the expansion and contraction of the Sc–OH bond lengths, respectively). Overall, the calculated ^27^Al and ^45^Sc *δ*_iso_ and *C*_*Q*_ values for a given composition do not change significantly between all four pore forms, making it potentially difficult to distinguish between them using ^27^Al or ^45^Sc NMR spectroscopy. However, it is clear that the complex spectral lineshapes that will result (particularly for two-dimensional MQMAS experiments)^28^ contain detailed information on the local geometry.

## Conclusions

Compositionally complex materials are of increasing interest in chemistry, potentially enabling fine tuning of properties for specific applications. However, the results presented here highlight the challenges that may be encountered in studying the framework composition, cation disorder and breathing behaviour of (Al,Sc)-MIL-53, owing to the different pore forms that might be adopted by the calcined materials. The high energy of the OP form suggest that this might be more difficult to produce, and it may be that this will only be seen immediately after calcination (*i.e.*, before any hydration) where much higher temperatures are used. This was the case for (Al,Ga)-MIL-53, where calcined materials adopted primarily OP forms, but after rehydration, subsequent dehydration at lower temperatures produced mixed pore forms and increasing amounts of NP material with increasing Ga^3+^ content. The relative energy of OP (Al,Sc)-MIL-53 decreases with increasing Al^3+^ content and so it may be that materials with larger amounts of Al^3+^ are more likely to favour this form upon heating, while those with higher Sc^3+^ content will preferentially adopt the more closed HT, LT or NP forms, particularly after dehydration. The HT, LT and NP forms have similar energies, but the LT form will likely be favoured at higher Sc content and the NP form at higher Al content. Interestingly, the relative energies of these forms also vary with the cation arrangement (*i.e.*, into chains, layers or more random arrangements), which may lead to mixed pore forms for different crystallites even they have similar compositions.

The mixing enthalpies for all calcined mixed-metal (Al,Sc)-MIL-53 materials are shown to be positive, largely because of the larger size difference between Al^3+^ and Sc^3+^ (at least when compared to Al^3+^ and Ga^3+^), although the free energy differences will also be affected by entropic contributions. It should also be noted that these materials are not directly prepared in these calcined dehydrated forms, but in an as-made form, which will determine the feasibility and likely cation ordering. However, the results do suggest care might have to be taken when calcining and/or dehydrating the materials to avoid framework decomposition.

The predicted NMR parameters show differences for all four forms of (Al,Sc)-MIL-53, suggesting this might be a potentially useful approach to follow breathing behaviour, although the presence of mixed pore materials may lead to spectral overlap. Many of the parameters also show a dependence on composition, offering possibilities for determination of the framework composition and any preferences for ordering or clustering of cations. It is clear each nuclide that can be studied has the potential to provide information (with different advantages and disadvantages for each pore form), and it may be best to combine approaches to obtain the most detailed information. However, it was seen that ^17^O NMR spectroscopy is particularly useful, with good resolution of the different types of μ_2_-OH groups (*i.e.*, X–O(H)–Y), which may enable the framework composition and ordering to be obtained from simple MAS experiments, rather than requiring very high fields, or the use of lengthy and non-quantitative MQMAS experiments. Distinct differences in the position of MQMAS lineshapes for carboxylate oxygens (both in terms of the nature of the bonded cation and the type of pore form adopted) were also predicted, which may provide further support to the structural conclusions. However, the requirement to isotopically enrich in ^17^O will add extra complexity to the synthetic challenge, with the need for cost-effective and atom-efficient enrichment methods limiting the synthetic approaches possible if enrichment is carried out during the initial synthesis. Although post-synthetic enrichment is also possible, this results in additional steps/transformations and may limit the access to particular pore forms.

The ability to fine tune the physical and chemical properties of MOFs offers a great opportunity to think about the rational design of materials. The use of computation prior to challenging and (for ^17^O enriched materials) expensive synthetic work provides insight into the possible behaviour that might be observed and the ability to evaluate the solid-state NMR spectroscopic approaches that may be best for providing the information needed. Although beyond the scope of the current study, it may also be interesting and insightful to consider the possible hydrated forms of (Al,Sc)-MIL-53 and, if possible, possible models for the as-made materials. The use of ensemble-based modelling approaches enables a complete set of symmetry unique structures to be considered and will, ultimately, enable the simulation of the complete NMR spectrum for fixed compositions for comparison to experiment.

## Author contributions

The manuscript was written through contributions of all authors. All authors have given approval to the final version of the manuscript.

## Conflicts of interest

There are no conflicts to declare.

## Supplementary Material

CP-025-D3CP04147F-s001
