# Supplementary material for: Computational NMR investigation of mixed-metal (Al,Sc)-MIL-53 and its phase transitions
Source: Phys Chem Chem Phys. 2023 Sep 22;25(39):26486–96. doi: 10.1039/d3cp04147f (PMC10566452; doi:10.1039/d3cp04147f)
Supplement: CP-025-D3CP04147F-s001 [file CP-025-D3CP04147F-s001.pdf]

Electronic Supplementary Material (ESI) for Physical Chemistry Chemical Physics.
This journal is © the Owner Societies 2023

Computational NMR Investigation of Mixed-Metal (Al,Sc)-MIL-53 and its Phase Transitions

Zachary H. Davis, Emma A. L. Borthwick, Russell E. Morris and Sharon E. Ashbrook

School of Chemistry, EaStCHEM and Centre of Magnetic Resonance, University of St Andrews, St Andrews, KY16 9ST, United Kingdom

Supporting Information

S1. Structure generation

S2. Calculation of the reference shielding

S3. Comparison of results from different versions of CASTEP

S4. Calculated unit cell volumes

S5. Calculation of degeneracy-weighted E_{mix}

S6. Additional calculated NMR parameters

S7. Calculation of δ_1 and δ_2 for a I = 5/2 3QMAS NMR spectrum

S8. References

S1. Structure generation

Structural models for ($Al_{1-x}Sc_x$)-MIL-53 in the OP, NP, HT and LT forms were generated from crystal structure data reported in literature.[S1,S2] Hydrogen atoms were added to the OP, HT and LT structures to produce aryl and hydroxyl groups where appropriate. The NP model was based on the theoretical structure produced in work of Bignami *et al.*,[S3] where an experimentally derived hydrated structure of Al-MIL-53 was optimised before removal of water modules and subsequent re-optimisation. Supercells (1 × 1 × 2) were generated for the OP and HT forms, such that eight metal sites were present in the unit cell, matching the number present in the NP and LT forms. For NP, HT and LT structures, the unit cells were translated to enable direct comparison with the OP structures using the transformation matrices T_{NP}, T_{HT} and T_{LT} where

$$T_{NP} = \begin{pmatrix} 0 & 0 & 1 \\ 1 & 0 & 0 \\ 0 & 1 & 0 \end{pmatrix}, \tag{S1.1}$$

$$T_{HT} = \begin{pmatrix} 0 & 0 & \bar{1} \\ \bar{1} & 0 & 0 \\ 0 & 1 & 0 \end{pmatrix}, \tag{S1.2}$$

$$T_{LT} = \begin{pmatrix} 0 & 1 & 0 \\ 1 & 0 & 0 \\ 0 & 0 & 1 \end{pmatrix}. \tag{S1.3}$$

These transformations ensured that the definition of unit cell axes were comparable between the four model systems. Figure S1.1 shows the resulting unit cell structures and numbering scheme used for the eight metal sites. Numbering of the cation sites is consistent between all four of the unit cells (but note that this does not indicate crystallographic inequivalence in the end member).

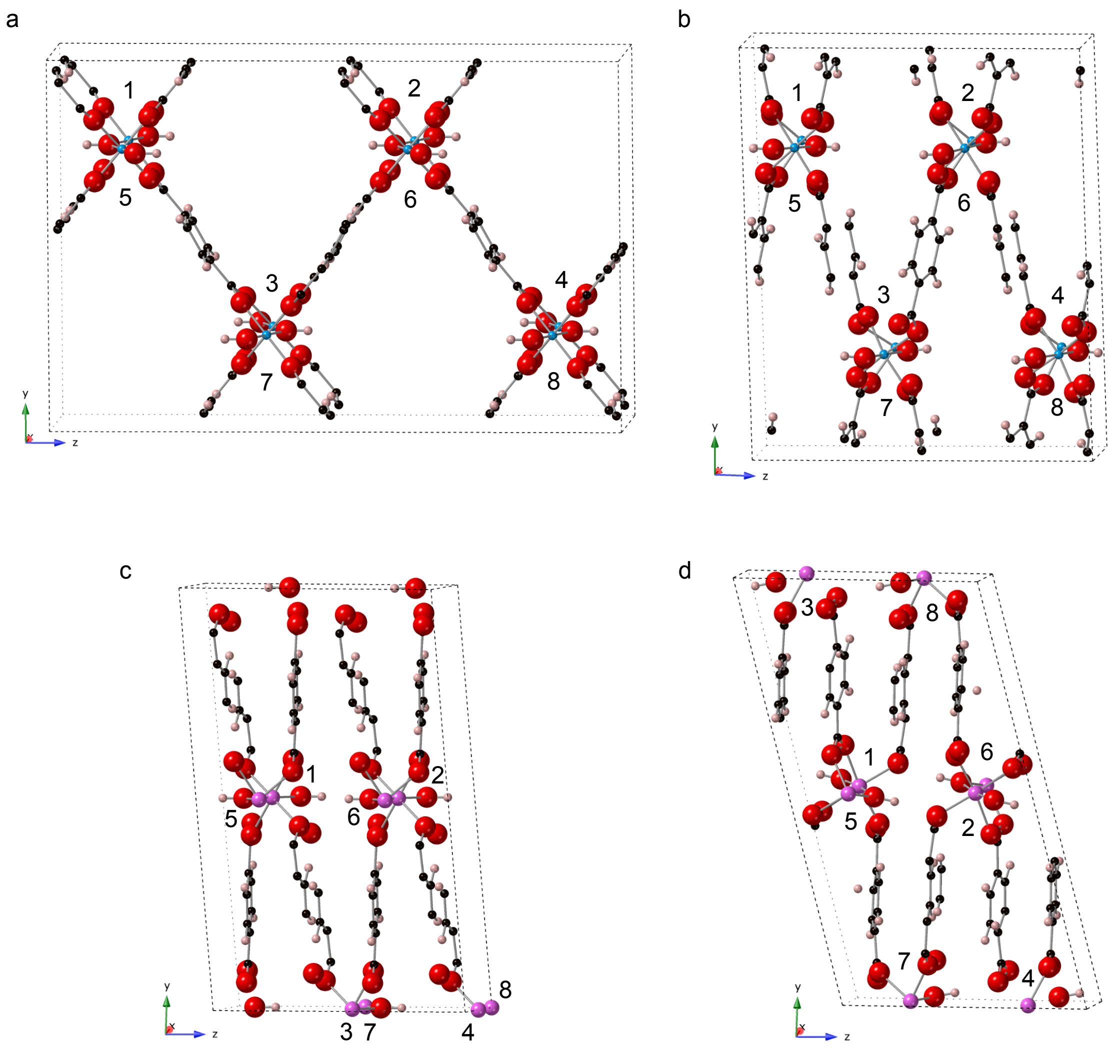

Figure S1.1 Schematic showing unit cell structures used for generating models for DFT calculations of (a) OP, (b) NP, (c) HT and (d) LT MIL-53 structures, with metal site numbering used to generate mixed-metal composition and substitution patterns.

As described in the main text, the SOD program[S4] was used to generate all possible symmetry inequivalent cation arrangements for each pore form based on their space groups: OP (*Imma*), NP ($P2_1/c$), HT ($C2/c$) and LT ($P2_1/c$). These space groups result in one, three, one and two crystallographically unique cation sites within the OP, NP, HT and LT unit cells, respectively, and 34, 88, 76 and 32 unique cation arrangements for the set of compositions explored (including the Al- and Sc-MIL-53 end members). Table S1.1 gives the complete set of $(Al_{1-x}Sc_x)$-MIL-53 structural models studied computationally, and information on the cation arrangement, composition, and configurational degeneracies. With the exception of the end members, the substitution pattern for a given structure is contained within the structure name. The prefix denotes

which type of cation is located at the given numbered positions. For example, Sc-18 contains Sc^{3+} on sites 1 and 8, and the remaining sites contain Al^{3+}, etc. Table S1.1 also describes the "type" or ordering, i.e., whether the cations are best described as being situated in layers (L) or chains (C), are found in structures that are end members or have very low levels of substitution (E) or are found in structures that have overall less ordering (O).

Table S1.1 Compositions, substitution patterns and configurational degeneracies of the structural models of OP, NP, HT and LT ($Al_{1-x}Sc_x$)-MIL-53 (generated by SOD[S4]) studied by DFT.

Structure name	Configurational degeneracy				x	Type[a]	Parent OP/HT structure[b]
	NP	LT	HT	OP			
Al-MIL-53	1	1	1	1	1	E	-
Al-1	4	4	8	8	0.125	E	-
Al-3	2	4	-	-	0.125	E	Al-1
Al-8	2	-	-	-	0.125	E	Al-1
Al-12	2	2	4	4	0.25	L	-
Al-13	4	4	8	8	0.25	L	-
Al-15	2	2	-	-	0.25	C	Al-48
Al-16	2	2	4	4	0.25	O	-
Al-17	4	4	8	8	0.25	O	-
Al-18	4	4	-	-	0.25	O	Al-17
Al-38	1	-	-	-	0.25	O	Al-16
Al-47	1	2	-	-	0.25	O	Al-16
Al-48	2	2	4	4	0.25	C	-
Al-58	4	4	-	-	0.25	L	Al-13
Al-78	2	2	-	-	0.25	L	Al-12
Al-123	2	4	8	8	0.375	L	-
Al-124	2	-	-	-	0.375	L	Al-123
Al-125	4	4	8	8	0.375	C	-
Al-127	2	-	-	-	0.375	O	Al-278
Al-134	4	4	-	-	0.375	L	Al-123
Al-135	4	4	16	16	0.375	C	-

Al-136	4	4	16	16	0.375	O	-
Al-137	4	4	-	-	0.375	C	Al-135
Al-138	4	4	-	-	0.375	O	Al-136
Al-147	4	4	-	-	0.375	O	Al-136
Al-148	4	4	-	-	0.375	C	Al-135
Al-158	4	4	-	-	0.375	C	Al-135
Al-168	4	4	-	-	0.375	O	Al-136
Al-278	4	4	8	8	0.375	O	-
Al-347	2	4	-	-	0.375	C	Al-125
Al-356	2	4	-	-	0.375	O	Al-278
Al-378	2	-	-	-	0.375	C	Al-125
Sc-1234	2	2	2	2	0.5	L	-
Sc-1235	4	4	16	16	0.5	C	-
Sc-1236	4	4	-	-	0.5	C	Sc-1235
Sc-1237	2	4	8	8	0.5	C	-
Sc-1247	2	4	8	8	0.5	L	-
Sc-1256	1	1	2	2	0.5	C	-
Sc-1257	4	4	16	16	0.5	C	-
Sc-1278	2	2	2	2	0.5	O	-
Sc-1345	4	4	-	-	0.5	C	Sc-1237
Sc-1346	4	4	-	-	0.5	L	Sc-1247
Sc-1347	4	4	-	-	0.5	C	Sc-1235
Sc-1357	4	2	4	4	0.5	C	-
Sc-1358	2	4	8	8	0.5	C	-
Sc-1367	4	4	-	-	0.5	C	Sc-1358
Sc-1368	2	2	4	4	0.5	O	-
Sc-1457	2	-	-	-	0.5	C	Sc-1358
Sc-2347	4	4	-	-	0.5	C	Sc-1235
Sc-2367	-	2	-	-	0.5	C	Sc-1357
Sc-2356	4	4	-	-	0.5	C	Sc-1257
Sc-2358	2	2	-	-	0.5	O	Sc-1368
Sc-3457	4	4	-	-	0.5	C	Sc-1257
Sc-3458	4	4	-	-	0.5	C	Sc-1257
Sc-3478	1	1	-	-	0.5	C	Sc-1256

Sc-3567	2	-	-	-	0.5	C	Sc-1237
Sc-4567	2	-	-	-	0.5	L	Sc-1247
Sc-123	2	4	8	8	0.675	L	-
Sc-124	2	-	-	-	0.675	L	Sc-123
Sc-125	4	4	8	8	0.675	C	-
Sc-127	2	-	-	-	0.675	O	Sc-278
Sc-134	4	4	-	-	0.675	L	Sc-123
Sc-135	4	4	16	16	0.675	C	-
Sc-136	4	4	16	16	0.675	O	-
Sc-137	4	4	-	-	0.675	C	Sc-135
Sc-138	4	4	-	-	0.675	O	Sc-136
Sc-147	4	4	-	-	0.675	O	Sc-136
Sc-148	4	4	-	-	0.675	C	Sc-135
Sc-158	4	4	-	-	0.675	C	Sc-135
Sc-168	4	4	-	-	0.675	O	Sc-136
Sc-278	4	4	8	8	0.675	O	-
Sc-347	2	4	-	-	0.675	C	Sc-125
Sc-356	2	4	-	-	0.675	O	Sc-278
Sc-378	2	-	-	-	0.675	C	Sc-125
Sc-12	2	2	4	4	0.75	L	-
Sc-13	4	4	8	8	0.75	L	-
Sc-15	2	2	-	-	0.75	C	Sc-48
Sc-16	2	2	4	4	0.75	O	-
Sc-17	4	4	8	8	0.75	O	-
Sc-18	4	4	-	-	0.75	O	Sc-17
Sc-38	1	-	-	-	0.75	O	Sc-16
Sc-47	1	2	-	-	0.75	O	Sc-16
Sc-48	2	2	4	4	0.75	C	-
Sc-58	4	4	-	-	0.75	L	Sc-13
Sc-78	2	2	-	-	0.75	L	Sc-12
Sc-1	4	4	8	8	0.825	E	-
Sc-3	2	4	-	-	0.825	E	Sc-1
Sc-8	2	-	-	-	0.825	E	Sc-1
Sc-MIL-53	1	1	1	1	0	E	-

[a] L = layers, C = chains, E = end member/low mixing and O = less ordering.

[b] The equivalent OP and HT cation arrangement after removing symmetry restraints from the given NP or LT unit cell.

S2. Calculation of the reference shielding

Reference shieldings (σ_{ref}) were determined for all nuclei present (i.e., ^{1}H, ^{13}C, ^{17}O, ^{27}Al and ^{45}Sc) by comparing calculated values of σ_{iso} to experimental values of δ_{iso} for a model system, with

$$\sigma_{iso} = m\,\delta_{iso} + \sigma_{ref} \,, \tag{S2.1}$$

where m is the gradient, sometimes referred to as a scaling factor, and σ_{ref} is determined from the y intercept. For ^{1}H, ^{13}C, ^{17}O and ^{27}Al, these values were obtained by comparison of the calculated and experimental NMR parameters for OP Al-MIL-53. Plots of calculated σ_{iso} against experimental δ_{iso} for ^{1}H, ^{13}C and ^{17}O are shown in Figure S2.1. Using Equation S2.1, values for the gradient, m, and the reference shielding, σ_{ref}, were calculated to be: −1.117 and 30.873, respectively, for ^{1}H; −0.958 and 163.59, respectively, for ^{13}C; and −1.105 and 272.16, respectively, for ^{17}O. For ^{27}Al, a single peak is observed in the spectrum with δ_{iso} = 3 ppm, and therefore the average of the eight ^{27}Al calculated σ_{iso} values was taken (for the eight metal sites in the unit cell of Al-MIL-53), giving σ_{ref} = 556.42 ppm. For ^{45}Sc, σ_{ref} = 772.85 ppm was used based on experimental ^{45}Sc δ_{iso} values (54.7 ppm and 56.5 ppm) for LT Sc-MIL-53.[S2] Note that while differences in absolute values (and small differences in relative values) may be expected if calculations are referenced differently (e.g., using different model materials or a wider set of materials) these will not affect the overall trends and conclusions outlined in the main text.

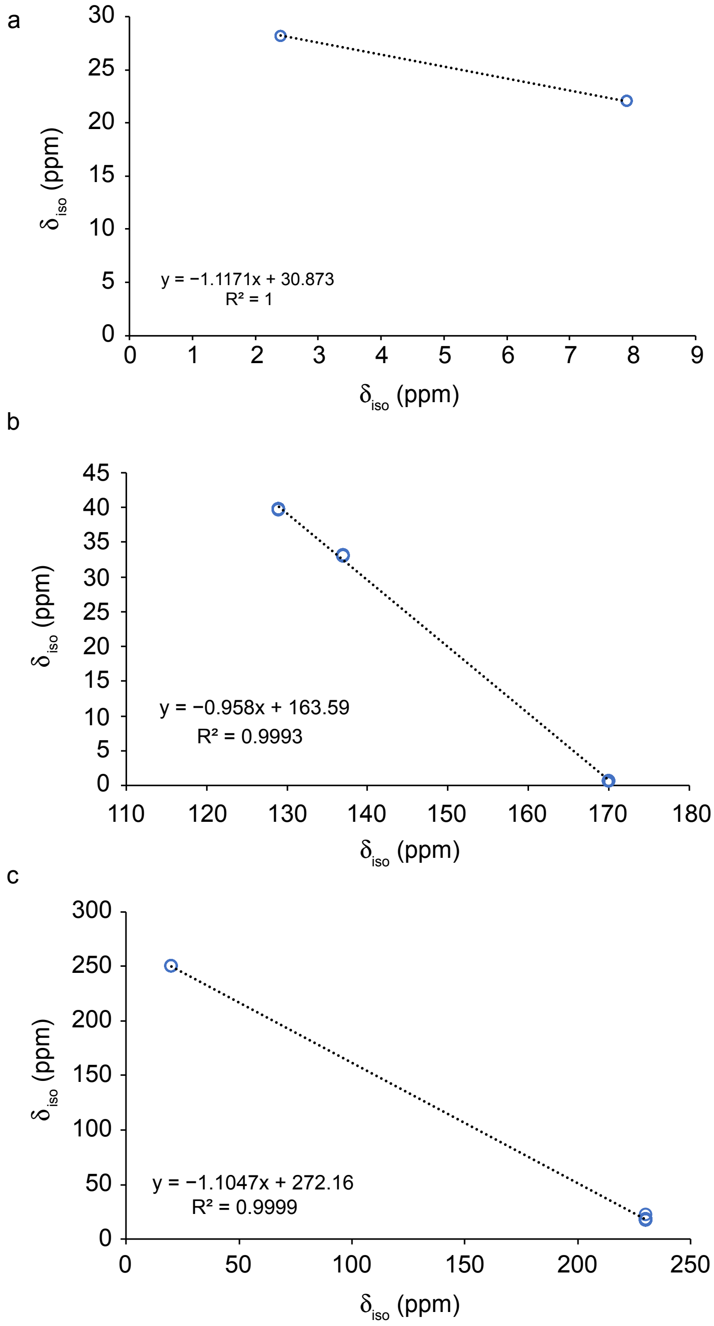

Figure S2.1 Plots of calculated σ_{iso} against experimental δ_{iso} for OP Al-MIL-53 and corresponding lines of best fit for (a) ^{1}H, (b) ^{13}C and (c) ^{17}O.

S3. Comparison of results from different versions of CASTEP

As described in the methods section of the main text, calculations have been carried out using CASTEP[S5-S8] versions 18.1 and 19.11. Comparisons of σ_{iso} and, where applicable, C_Q values for the calculated ^{1}H, ^{13}C, ^{17}O and ^{27}Al NMR parameters in the HT and LT forms of Al-MIL-53 determined using CASTEP 18.1 and CASTEP 19.11 are shown in Figures S3.1-S3.6. These plots show the calculated σ_{iso} and C_Q values for the two codes typically agree well with one another (as expected), and where deviations do occur these are typically small, such as for the calculated ^{27}Al C_Q values. This indicates that calculated parameters obtained using the two versions of the code are consistent and can be considered together.

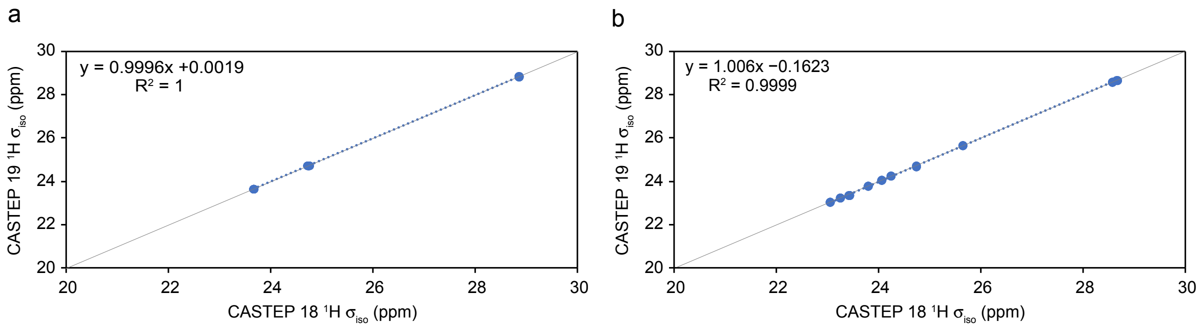

Figure S3.1 Plots showing calculated ^{1}H σ_{iso} values determined using CASTEP versions 18.1 and 19.11 for (a) HT and (b) LT Al-MIL-53. The grey lines indicate y = x.

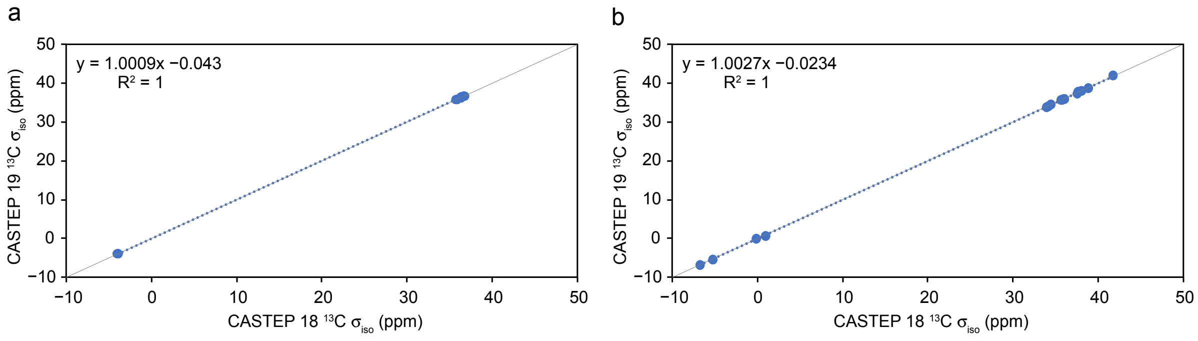

Figure S3.2 Plots showing calculated ^{13}C σ_{iso} values determined using CASTEP versions 18.1 and 19.11 for (a) HT and (b) LT Al-MIL-53. The grey lines indicate y = x.

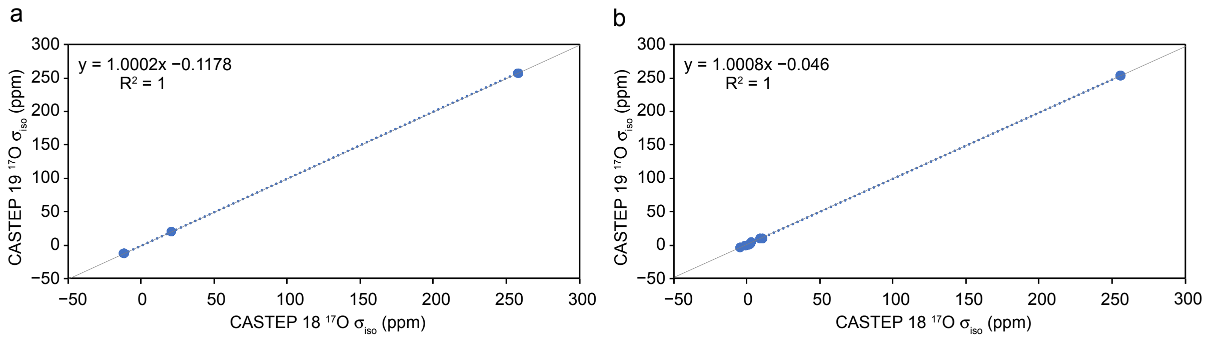

Figure S3.3 Plots showing calculated ^{17}O σ_{iso} values determined using CASTEP versions 18.1 and 19.11 for (a) HT and (b) LT Al-MIL-53. The grey lines indicate y = x.

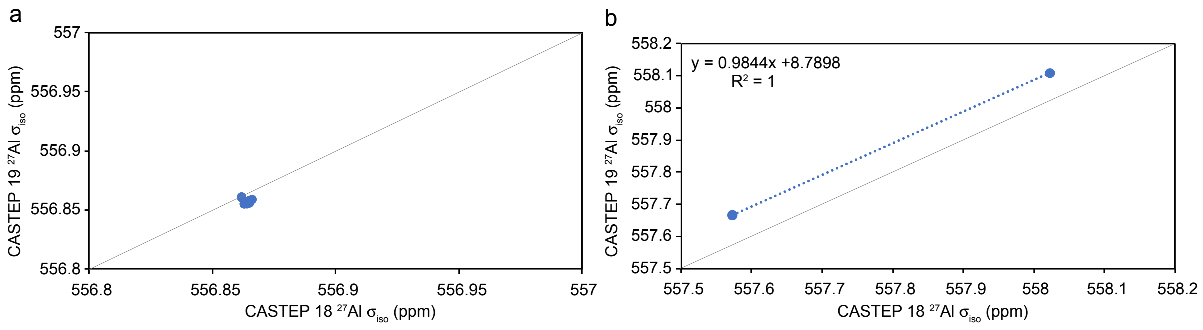

Figure S3.4 Plots showing calculated ^{27}Al σ_{iso} values determined using CASTEP versions 18.1 and 19.11 for (a) HT and (b) LT Al-MIL-53. The grey lines indicate y = x. Note the very small scales on both axes.

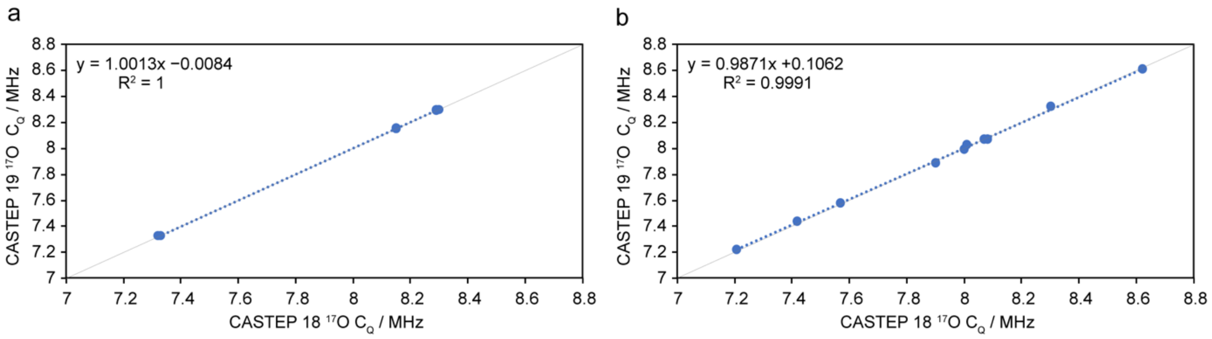

Figure S3.5 Plots showing calculated ^{17}O C_Q values determined using CASTEP versions 18.1 and 19.11 for (a) HT and (b) LT Al-MIL-53. The grey lines indicate y = x.

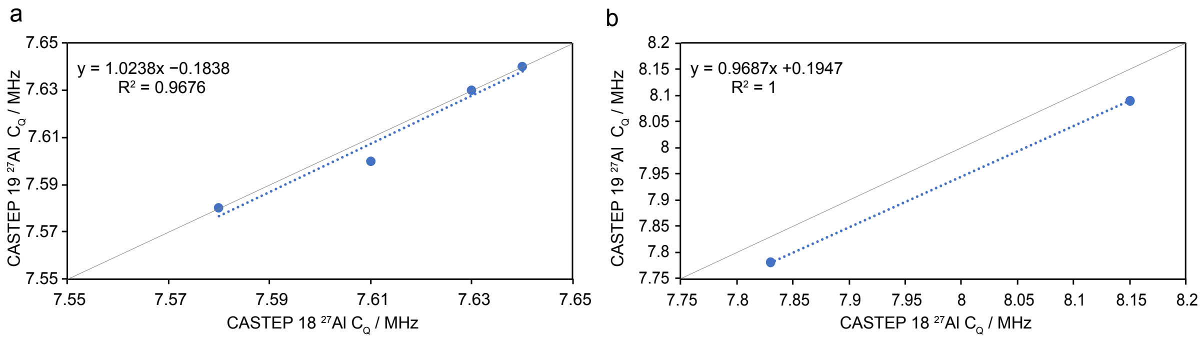

Figure S3.6 Plots showing calculated ^{27}Al C_Q values determined using CASTEP versions 18.1 and 19.11 for (a) HT and (b) LT Al-MIL-53. The grey lines indicate y = x. Note the very small scales on both axes.

S4. Calculated unit cell volumes

Figure S4.1 shows the calculated unit cell volume for the four different pore forms of ($Al_{1-x}Sc_x$)-MIL-53 as a function of the cation composition. In general, the unit cell volume increases with increasing x, likely because of the increased size of Sc^{3+} (0.745 Å) over Al^{3+} (0.535 Å).[S9] This volume increase is the largest for the OP form.

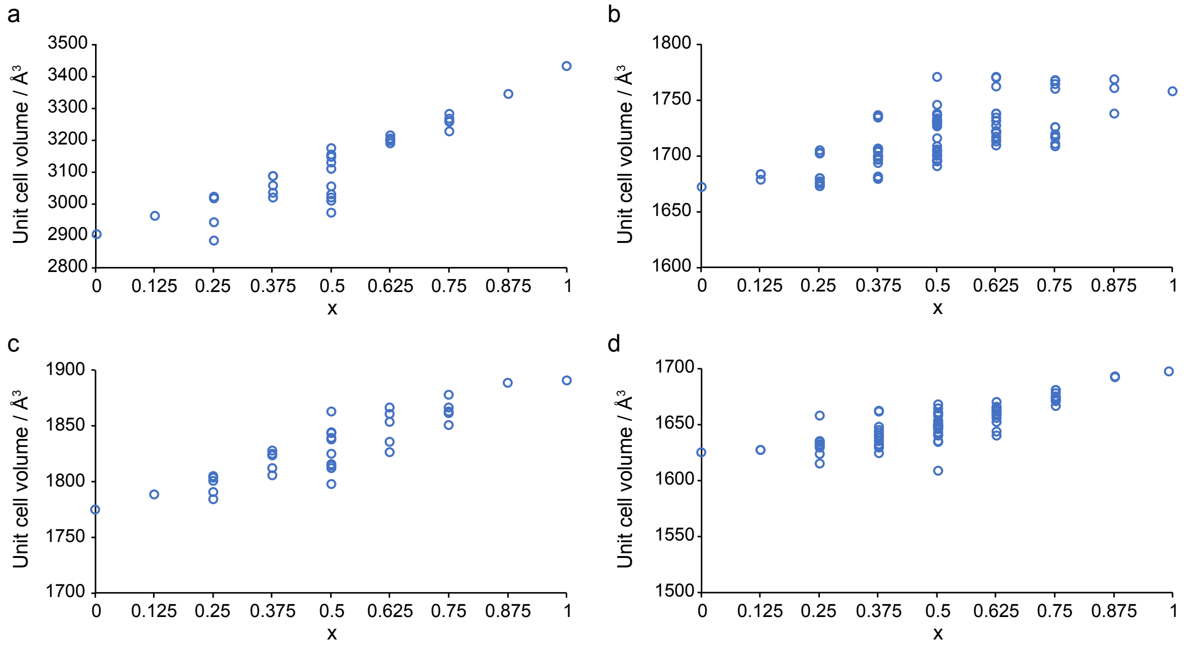

Figure S4.1 Plots showing the calculated unit cell volume as a function of the composition of the (a) OP, (b) NP (c) HT and (d) LT forms of ($Al_{1-x}Sc_x$)-MIL-53.

S5. Calculation of degeneracy-weighted E_{mix}

Figure S5.1 shows the calculated degeneracy-weighted E_{mix} values for the four pore forms of $(Al_{1-x}Sc_x)$-MIL-53 as a function of the cation composition. For all four forms E_{mix} is slightly more favourable (though still enthalpically unfavourable overall) for higher values of x. This suggests it is (relatively) more favourable to substitute the smaller Al^{3+} cation into a Sc-containing framework, rather than *vice versa*. The degeneracies for each structural model are provided in Table S1.1.

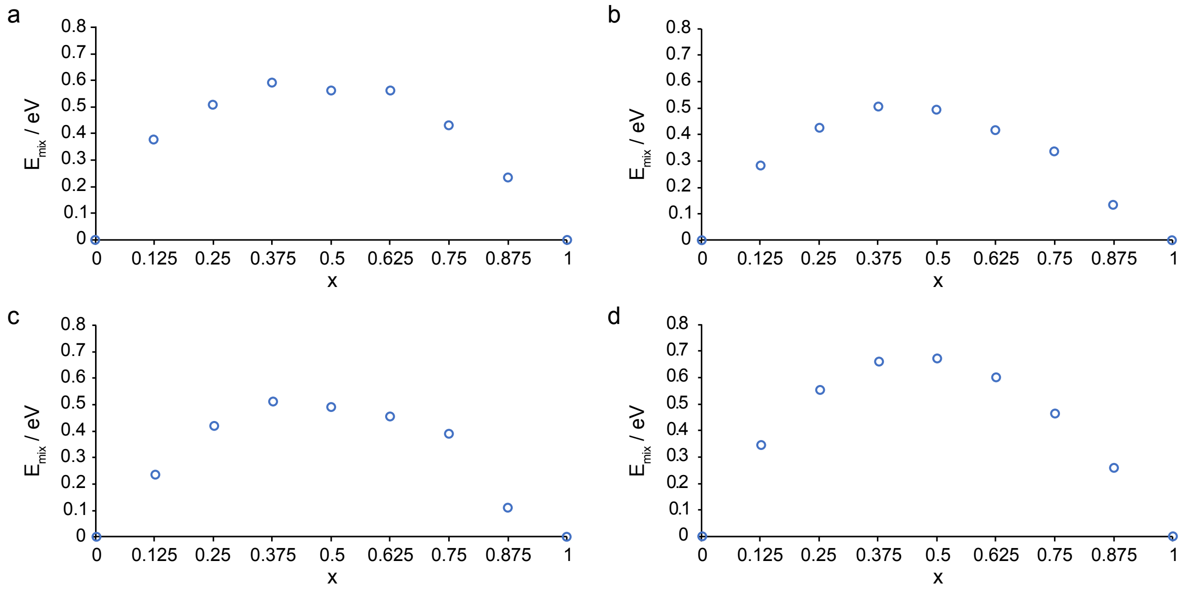

Figure S5.1 Plots of degeneracy-weighted mixing enthalpies for (a) OP, (b) NP, (c) HT and (d) LT $(Al_{1-x}Sc_x)$-MIL-53 as a function of composition.

S6. Additional calculated NMR parameters

Figure S6.1 shows the origin of the two unique C1 environments in LT ($Al_{1-x}Sc_x$)-MIL-53, $C1_a$ and $C1_b$ which arise due to the tilting of the MO_6 octahedra.

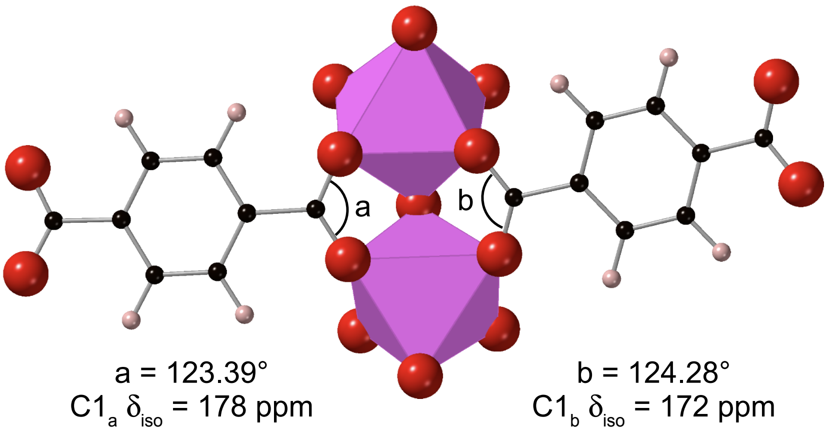

Figure S6.1 Schematic showing the two unique C1 environments in the LT form of ($Al_{1-x}Sc_x$)-MIL-53, shown here for x = 1. These two environments arise due to tilting of the MO_6 octahedra which affects the O-C1-O bond angle and results in different ^{13}C C1 δ_{iso} values.

Figure S6.2 shows plots of ^{1}H δ_{iso} as a function of composition for all four pore forms of ($Al_{1-x}Sc_x$)-MIL-53. Figure S6.3 shows plots of ^{2}H C_Q against δ_{iso} for all four pore forms of ($Al_{1-x}Sc_x$)-MIL-53.

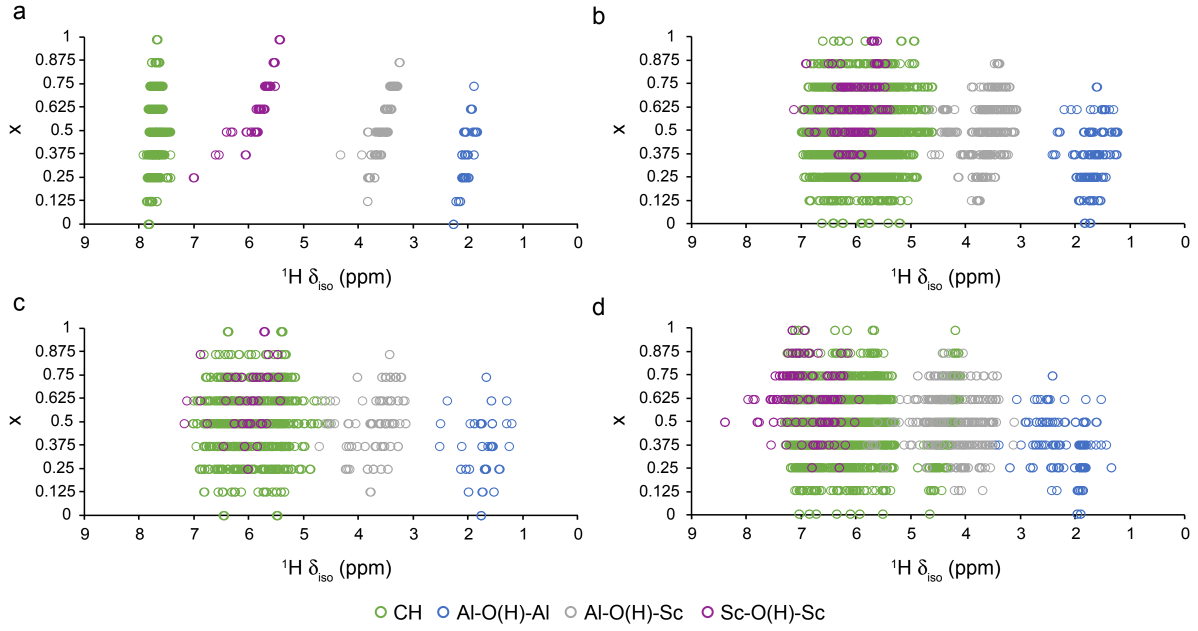

Figure S6.2 Plots showing calculated ^{1}H δ_{iso} for (a) OP, (b) NP, (c) HT and (d) LT ($Al_{1-x}Sc_x$)-MIL-53 as a function of composition, coloured by type of ^{1}H.

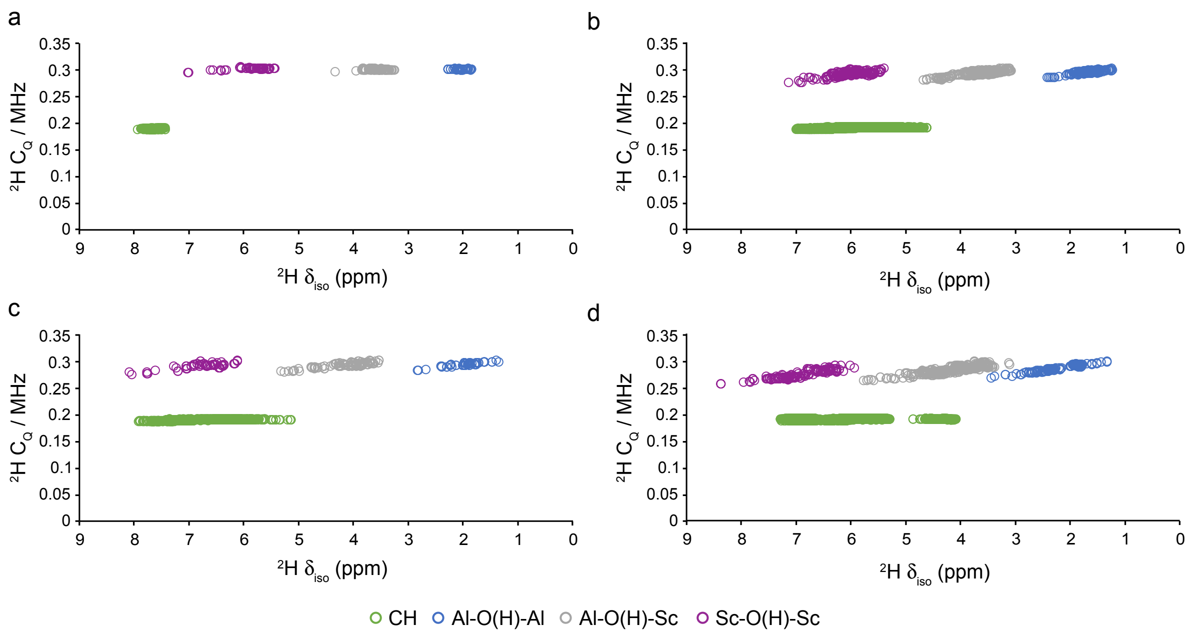

Figure S6.3 Plots showing calculated ^{2}H C_Q against δ_{iso} (a) OP, (b) NP, (c) HT and (d) LT $(Al_{1-x}Sc_x)$-MIL-53, coloured by type of ^{2}H.

Figure S6.4 shows the correlation between the calculated ^{17}O δ_{iso} value and the Sc-O-Sc angle for Sc-O(H)-Sc groups in OP $(Al_{1-x}Sc_x)$-MIL-53.

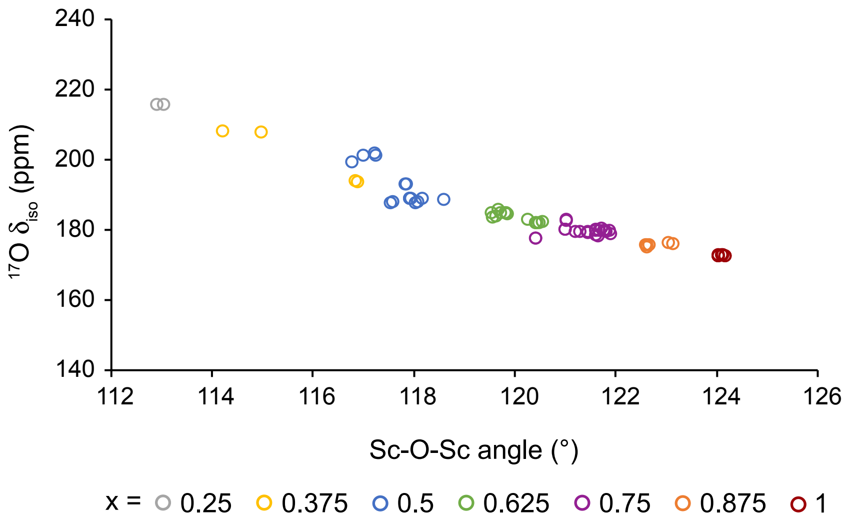

Figure S6.4 Plot showing calculated ^{17}O δ_{iso} values for Sc-O(H)-Sc groups in OP $(Al_{1-x}Sc_x)$-MIL-53 plotted as a function of the Sc-O-Sc angle, coloured by the composition of the overall framework.

Figure S6.5 shows a plot of calculated ^{17}O C_Q against δ_{iso} for oxygens in all four pore forms of $(Al_{1-x}Sc_x)$-MIL-53, in which distinct carboxylate groups can be seen for the NP, HT and LT forms.

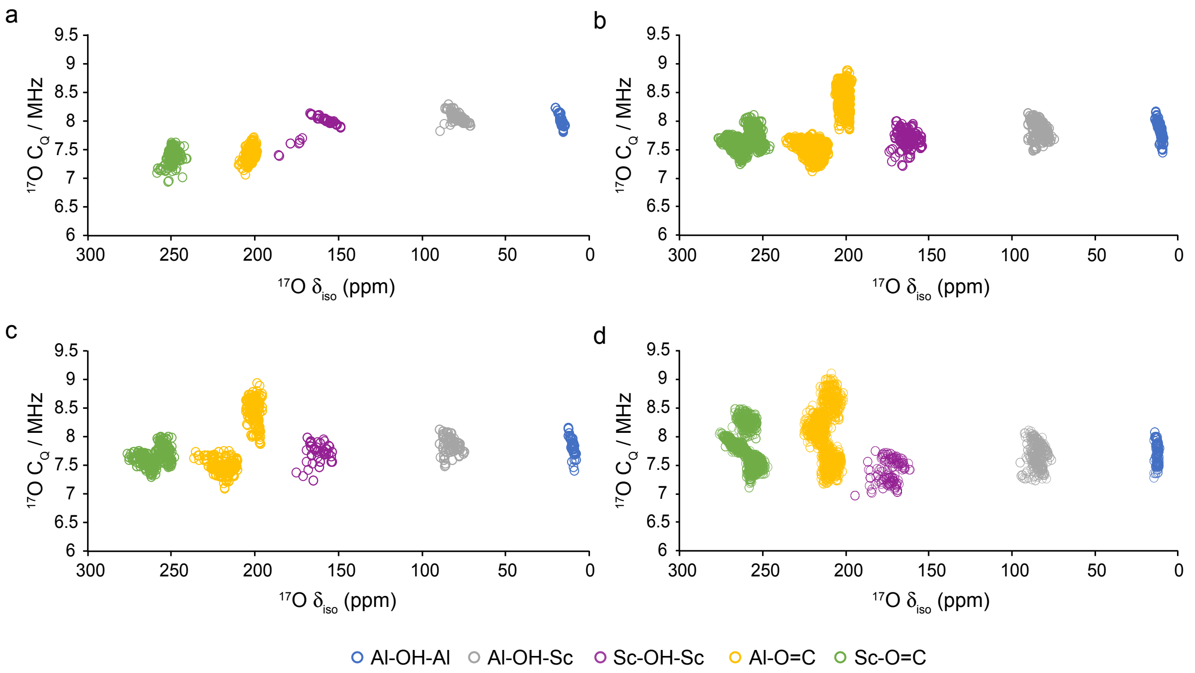

Figure S6.5 Plots of calculated ^{17}O C_Q against δ_{iso} for (a) OP, (b) NP, (c) HT and (d) LT $(Al_{1-x}Sc_x)$-MIL-53, coloured by the type of oxygen.

Figure S6.6 plots the calculated ^{17}O C_Q against δ_{iso} for carboxylate groups bonded to Sc^{3+} in LT $(Al_{1-x}Sc_x)$-MIL-53, showing variation with both the Sc-O bond length and Sc-O=C angle. No direct dependence is seen on the type of cation arrangement or composition.

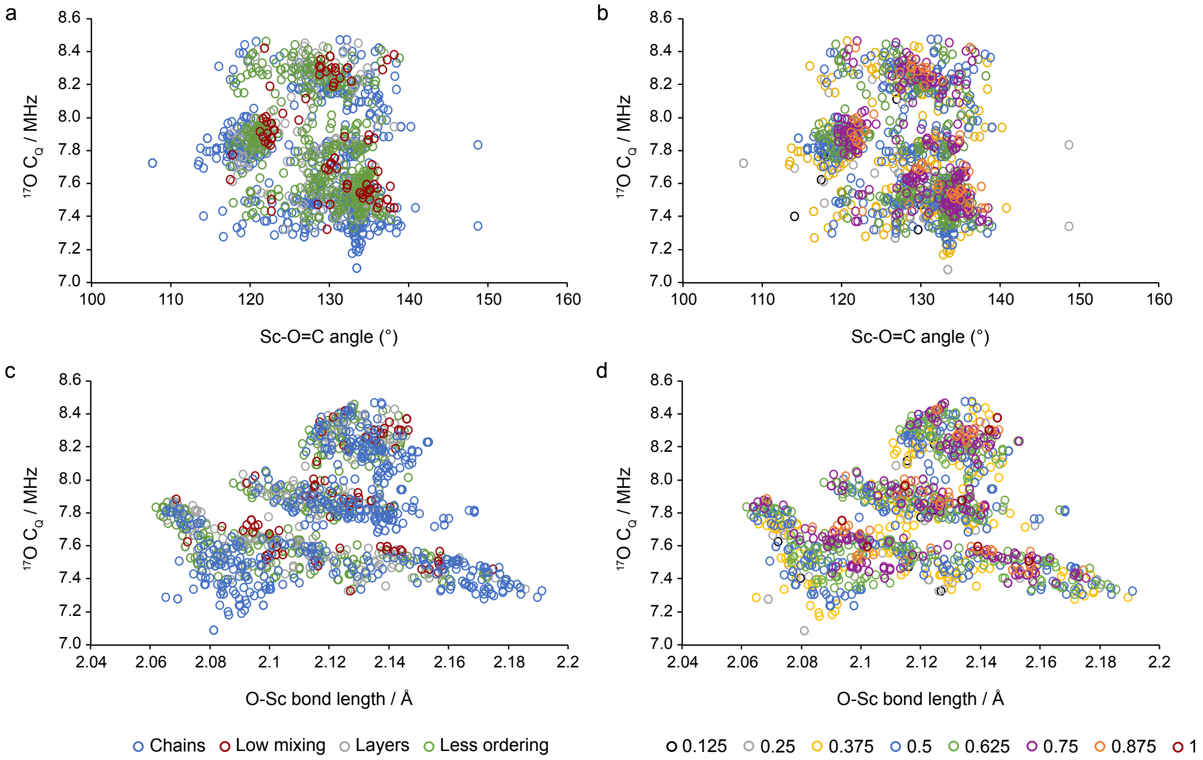

Figure S6.6 Plots of calculated ^{17}O C_Q values for Sc-O=C groups in LT $(Al_{1-x}Sc_x)$-MIL-53 plotted as a function of (a, b) the Sc-O=C angle and (c, d) the O-Sc bond length, coloured by (a, c) type of cation arrangement and (b, d) composition.

Figures S6.7 and 6.8 plot the calculated ^{17}O δ_{iso} and C_Q as a function of the distance between the carboxylate O and the hydrogen atom of the OH group across the pore for LT Sc-MIL-53 (Figure S6.7) and LT $(Al_{1-x}Sc_x)$-MIL-53 (Figure S6.8).

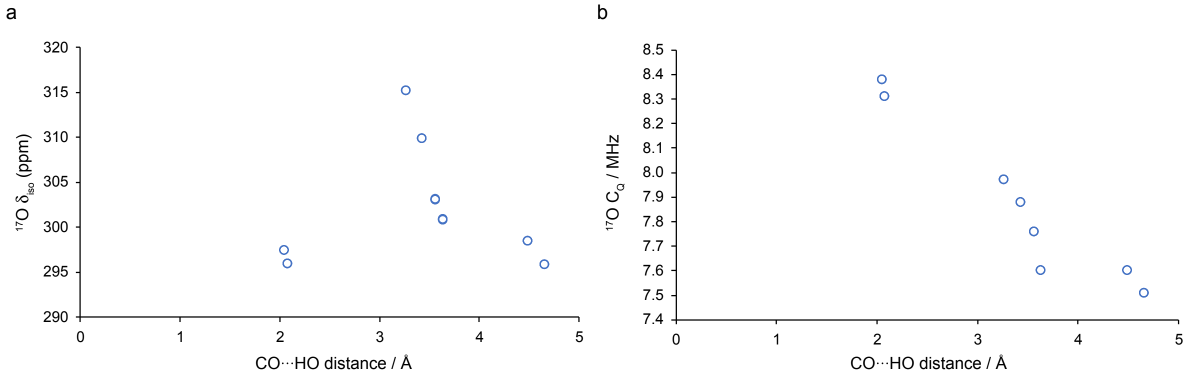

Figure S6.7 Plots of calculated ^{17}O (a) δ_{iso} and (b) C_Q values for Sc-O=C groups in LT Sc-MIL-53 plotted as a function of the distance between the carboxylate oxygen and the hydrogen atom of the OH group across the pore.

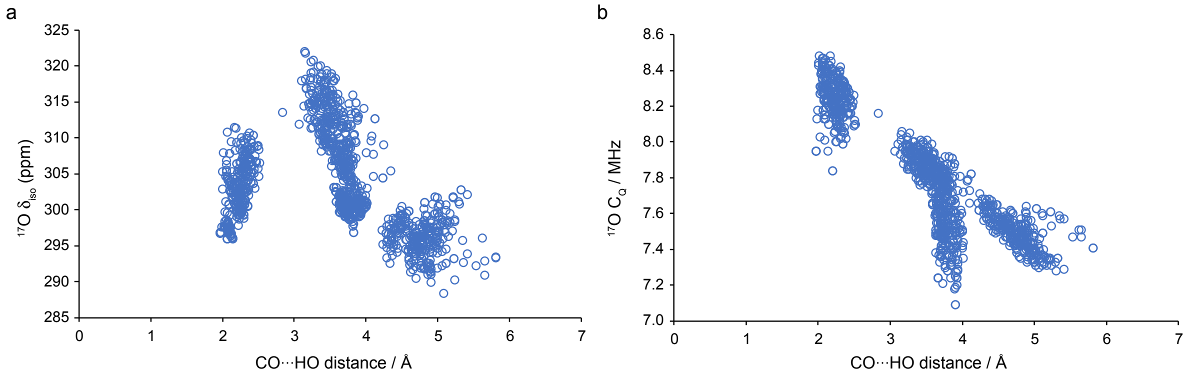

Figure S6.8 Plots of calculated ^{17}O (a) δ_{iso} and (b) C_Q values for Sc-O=C groups in LT $(Al_{1-x}Sc_x)$-MIL-53 (for all x) plotted as a function of the distance between the carboxylate oxygen and the hydrogen atom of the OH group across the pore.

Figures S6.9 and S6.10 plot the calculated δ_{iso} values for ^{27}Al and ^{45}Sc, respectively, for different compositions of $(Al_{1-x}Sc_x)$-MIL-53 for each of the four pore forms. For each nucleus two different types of cations site are seen, depending on the nature of the next nearest cations. (Note owing to the imposed ordering discussed in the main text these can only both be Al^{3+} or both be Sc^{3+}).

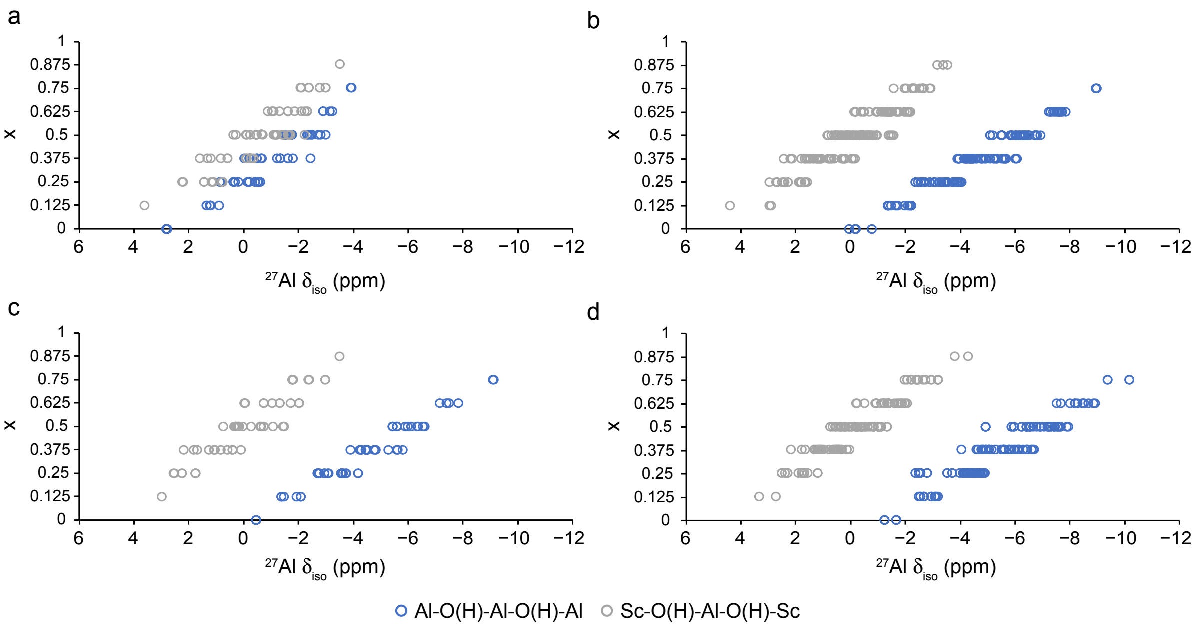

Figure S6.9 Plots showing calculated ^{27}Al δ_{iso} for (a) OP, (b) NP, (c) HT and (d) LT $(Al_{1-x}Sc_x)$-MIL-53 for varying compositions, coloured by the type of next nearest neighbouring metals.

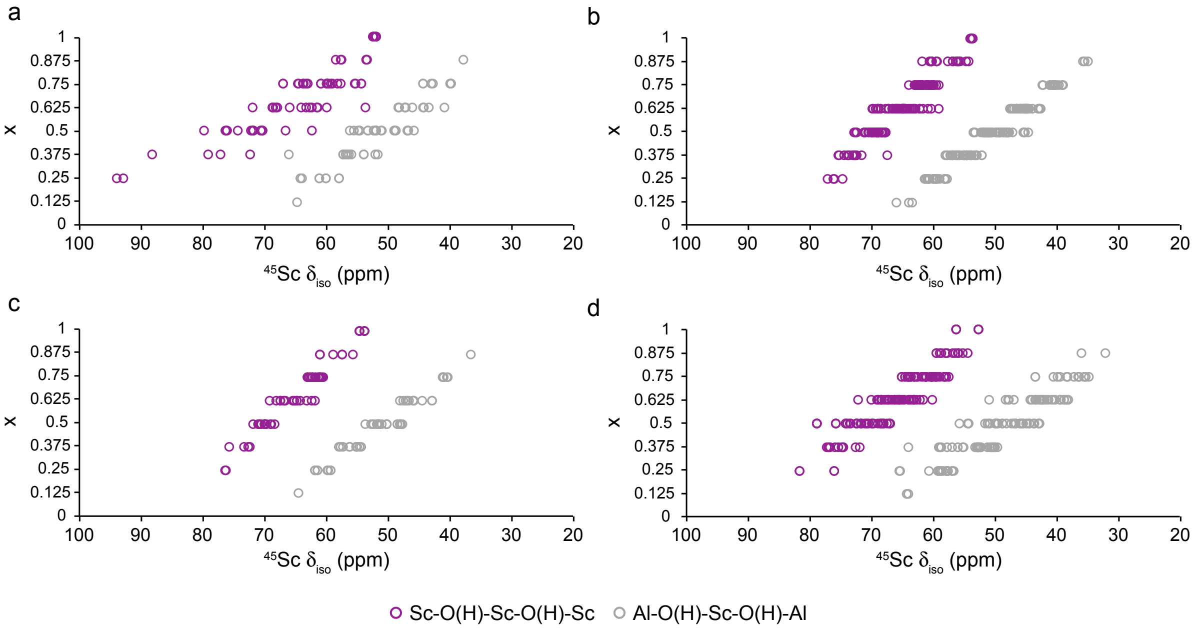

Figure S6.10 Plots showing calculated ^{45}Sc δ_{iso} for (a) OP, (b) NP, (c) HT and (d) LT ($Al_{1-x}Sc_x$)-MIL-53 for varying compositions, coloured by the type of next nearest neighbouring metals.

Figures S6.11 and S6.12 show plots of C_Q against δ_{iso} for ^{27}Al and ^{45}Sc, respectively, for all four pore forms of ($Al_{1-x}Sc_x$)-MIL-53.

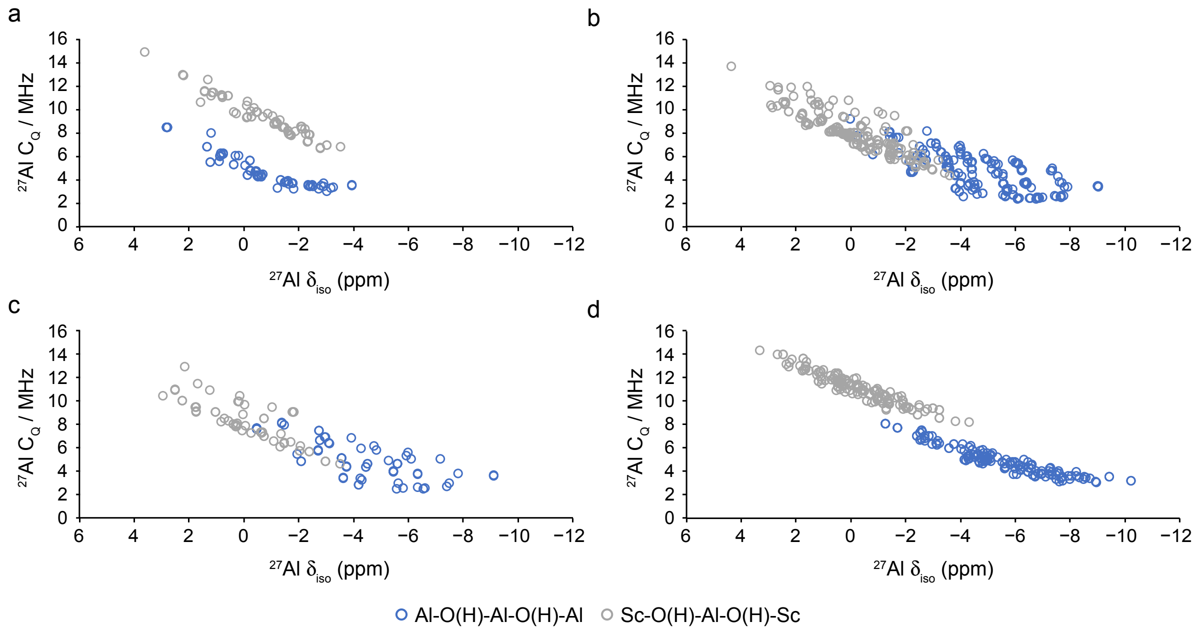

Figure S6.11 Plots of calculated ^{27}Al C_Q against δ_{iso} for (a) OP, (b) NP, (c) HT and (d) LT ($Al_{1-x}Sc_x$)-MIL-53, coloured by type of next nearest neighbouring metals.

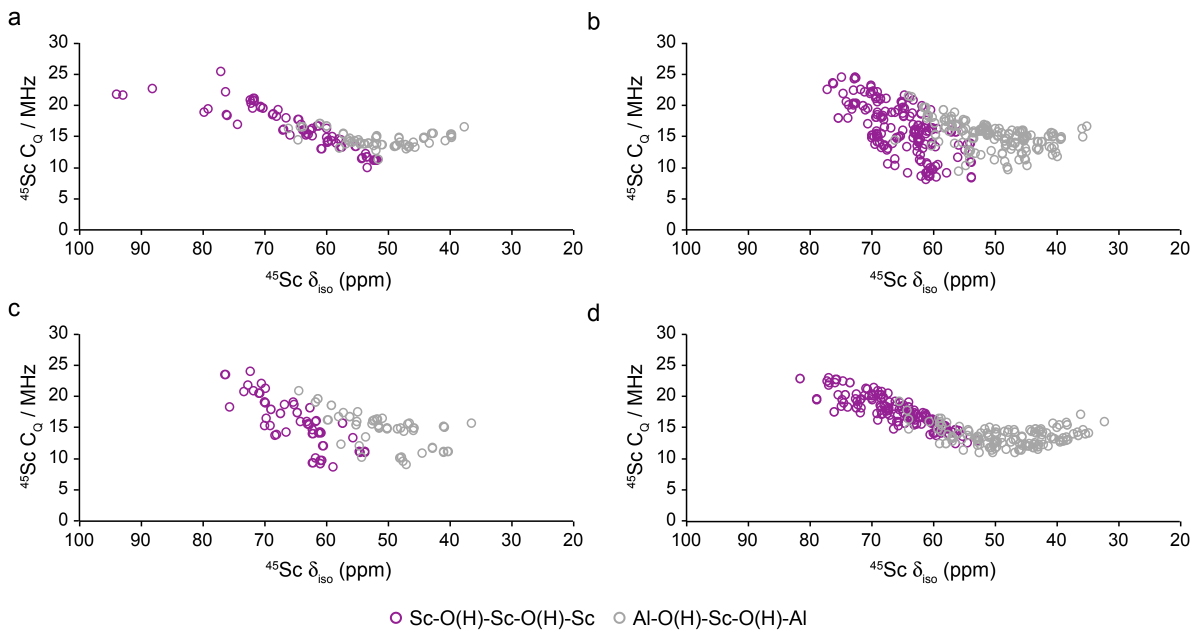

Figure S6.12 Plots of calculated ^{45}Sc C_Q against δ_{iso} for (a) OP, (b) NP, (c) HT and (d) LT ($Al_{1-x}Sc_x$)-MIL-53, coloured by type of next nearest neighbouring metals.

Figure S6.13 plots ^{45}Sc δ_{iso} as a function of the difference in the average Sc-OH and Sc-OC bond lengths (<Sc-OC> – <Sc-OH>) for a given ScO_6 octahedra for ($Al_{1-x}Sc_x$)-MIL-53 in the LT form coloured by the overall framework composition. These plots show a linear relationship between the two for both types of Sc^{3+}. Figure S6.14 shows the change in average Sc-OH bond length is greatest for Sc^{3+} in the Al-O(H)-Sc-O(H)-Al environment compared to that of Sc-O(H)-Sc-O(H)-Sc indicating greater structural variation around Sc^{3+} due to the presence of Al^{3+}, likely driven by differences in cation size distorting the framework structure.[S9]

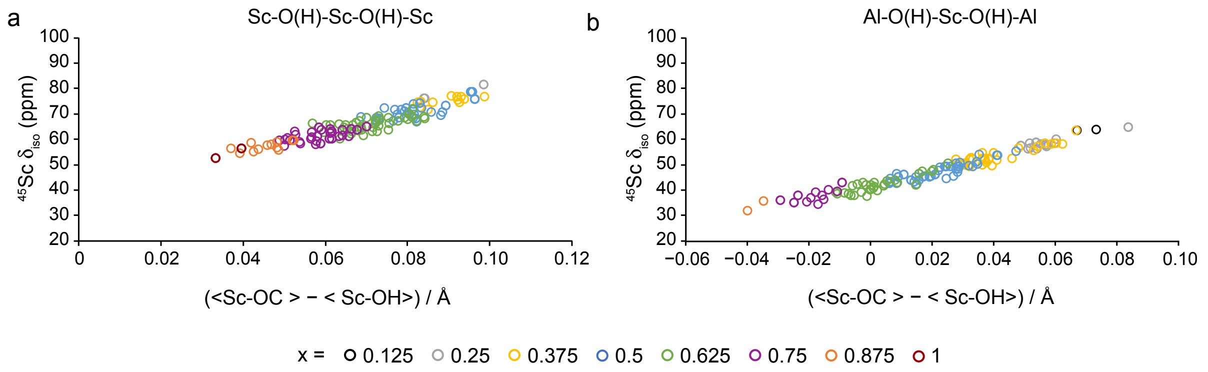

Figure 6.13 Plots of calculated ^{45}Sc δ_{iso} as a function of the difference in the average Sc-OH and Sc-OC bond lengths for the ScO_6 octahedra in LT $(Al_{1-x}Sc_x)$-MIL-53 for (a) Sc-O(H)-Sc-O(H)-Sc and (b) Al-O(H)-Sc-O(H)-Al. Points are coloured by composition of the overall framework.

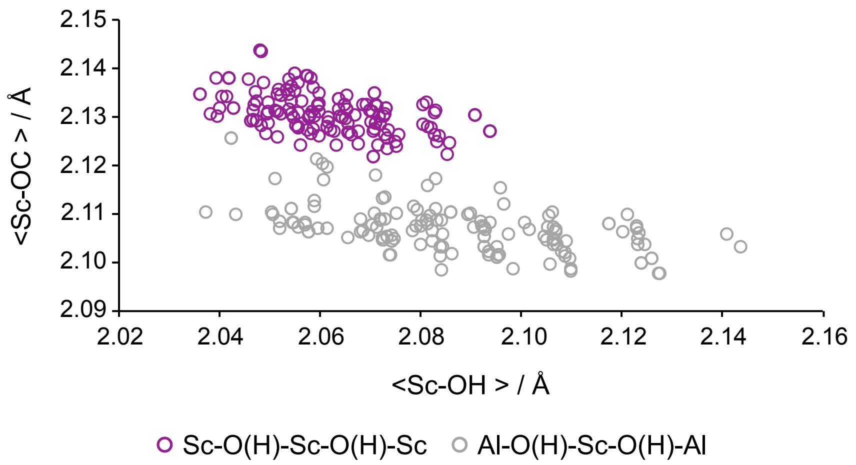

Figure 6.14 Plot of the average Sc-OC bond length against the average Sc-OH bond length for the ScO_6 octahedra in LT $(Al_{1-x}Sc_x)$-MIL-53, coloured by the type of next nearest neighbouring metals.

S7. Calculation of δ_1 and δ_2 for a I = 5/2 3QMAS NMR spectrum

The position of the centre-of-gravity (δ_1, δ_2) can be calculated (for a specified magnetic field) for a 3QMAS NMR spectrum for a spin I = 5/2, using the referencing convention specific in Ref. S10, with

$$\delta_1 = \frac{17}{31}\delta_{iso} + \frac{32}{96}\delta_Q \quad , \qquad (S7.1)$$

$$\delta_2 = \delta_{iso} - \frac{32}{96}\delta_Q \quad , \qquad (S7.2)$$

where δ_Q is

$$\delta_Q = \frac{3000}{40}\frac{P_Q}{\nu_0} \quad . \qquad (S7.3)$$

ν_0 is the Larmor frequency at the specific field and P_Q is defined as

$$P_Q = C_Q\sqrt{\left(1 + \frac{\eta_Q^2}{3}\right)} \quad , \qquad (S7.4)$$

where η_Q is the asymmetry of the quadrupolar tensor.[S11]

References

S1. T. Loiseau, C. Serre, C. Huguenard, G. Fink, F. Taulelle, M. Henry, T. Bataille and G. Férey, *Chem. Eur. J.*, 2004, **10**, 1373–1382.

S2. J. P. S. Mowat, V. R. Seymour, J. M. Griffin, S. P. Thompson, A. M. Z. Slawin, D. Fairen-Jimenez, T. Düren, S. E. Ashbrook and P. A. Wright, *Dalton Trans.*, 2012, **41**, 3937–3941.

S3. G. P. M. Bignami, Z. H. Davis, D. M. Dawson, S. A. Morris, S. E. Russell, D. McKay, R. E. Parke, D. Iuga, R. E. Morris and S. E. Ashbrook, *Chem. Sci.*, 2018, **9**, 850–859.

S4. R. Grau-Crespo, S. Hamad, C. R. A. Catlow and N. H. de Leeuw, *J. Phys. Condens. Matter*, 2007, **19**, 256201.

S5. S. J. Clark, M. D. Segall, C. J. Pickard, P. J. Hasnip, M. I. J. Probert, K. Refson and M. C. Payne, *Z. Für Krist. - Cryst. Mater.*, 2005, **220**, 567–570.

S6. C. J. Pickard and F. Mauri, *Phys. Rev. B*, 2001, **63**, 245101.

S7. J. R. Yates, C. J. Pickard and F. Mauri, *Phys. Rev. B*, 2007, **76**, 024401.

S8. M. Profeta, F. Mauri and C. J. Pickard, *J. Am. Chem. Soc.*, 2003, **125**, 541–548.

S9. R. D. Shannon, *Acta Crystallogr. A*, 1976, **32**, 751–767.

S10. K. J. Pike, R. P. Malde, S. E. Ashbrook, J. McManus and S. Wimperis, *Solid State Nucl. Magn. Reson.*, 2000, **16**, 203–215.

S11. S. E. Ashbrook, D. M. Dawson and J. M. Griffin, in *Local Structural Characterisation*, Eds. D. W. Bruce, D. O'Hare and R. I. Walton, John Wiley & Sons, Chichester, 2014.
